# Influence of a Darcy-Forchheimer porous medium on the flow of a radiative magnetized rotating hybrid nanofluid over a shrinking surface

**DOI:** 10.1038/s41598-021-03470-x

**Published:** 2021-12-20

**Authors:** Sumera Dero, Hisamuddin Shaikh, Ghulam Hyder Talpur, Ilyas Khan, Sayer O. Alharbim, Mulugeta Andualem

**Affiliations:** 1 Institute of Mathematics and Computer Science, University of Sindh, Jamshoro, 76080 Pakistan; 2 Department of Mathematics, Shah Abdul Latif University Khairpur Mirs, Sindh, Pakistan; 3 Department of Statistics, University of Sindh, Jamshoro, Pakistan; 4 Department of Mathematics, College of Science Al-Zulfi, Majmah University, Al-Majmah, 11952 Saudi Arabia; 5 Department of Mathematics, Bonga University, Bonga, Ethiopia

**Keywords:** Engineering, Mathematics and computing

## Abstract

In this paper, the heat transfer properties in the three-dimensional (3D) magnetized with the Darcy-Forchheimer flow over a shrinking surface of the $Cu + Al_{2} O_{3} /$ water hybrid nanofluid with radiation effect were studied. Valid linear similarity variables convert the partial differential equations (PDEs) into the ordinary differential equations (ODEs). With the help of the shootlib function in the Maple software, the generalized model in the form of ODEs is numerically solved by the shooting method. Shooting method can produce non-unique solutions when correct initial assumptions are suggested. The findings are found to have two solutions, thereby contributing to the introduction of a stability analysis that validates the attainability of first solution. Stability analysis is performed by employing if bvp4c method in MATLAB software. The results show limitless values of dual solutions at many calculated parameters allowing the turning points and essential values to not exist. Results reveal that the presence of dual solutions relies on the values of the porosity, coefficient of inertia, magnetic, and suction parameters for the specific values of the other applied parameters. Moreover, it has been noted that dual solutions exist in the ranges of $F_{s} \le F_{sc}$, $M \ge M_{C}$*,* $S \ge S_{C} ,$ and $K_{C} \le K$ whereas no solution exists in the ranges of $F_{s} > F_{sc}$, $M < M_{c}$, $S < S_{c}$, and $K_{C} > K$. Further, a reduction in the rate of heat transfer is noticed with a rise in the parameter of the copper solid volume fraction.

## Introduction

Recently, scholars are enrolled in the research of rotating flows within stretching and shrinking boundary layer problems because of their widespread use in rotor stator systems, food processing, cleaners of disk, spinning devices, gas turbine architecture, and many others. Wang ^1^ examined the flow of rotating fluid through the stretching sheet where momentum layer thickness was observed to decrease as the parameter of the rotational impact increased. Takhar et al.^2^ considered a rotating fluid flow on a stretched surface with the characteristics of a magnetic number. Shafique et al.^3^ investigated the rotating effect of the Maxwell fluid by considering binary chemical reactions and energy activation characteristics. They have found that “hydrodynamic boundary layer thins when rotation parameter λ is incremented. Oscillatory behavior in both *x*- and *y*-components of velocity is observed when rotation parameter λ is sufficiently large”. Rashad^4^ used mathematical modeling to check the effect of non-steady MHD flow of rotating fluid. Recently, Ullah et al.^5^ found that temperature and concentration were increasing functions of the porosity and the Forchheimer parameters during the examination of the rotating flow of the nanofluid. In addition, Hayat et al.^6^ extended the rotational flow model to examine the characteristics of homogeneous–heterogeneous nanofluid reactions. Lund et al.^7^ examined the 3D flow of rotating hybrid nanofluid over the exponential surface and found that the solution was not unique when the value of the rotating parameter was less than 0.1. Some important effects of the various physical parameter on the rotating flow can be seen in these articles^8–12^.

Energy sustainability and improvement of the performance of thermal devices are the focus of research in different industrial and engineering areas, such as power generation, microelectronics, and air-conditioning. A creative range of thermodynamics has been important for the sustainability of energy^13^. The systems of cooling work on a fluid flow by a force diffusion in the presence and absence of convective transfer of heat during such engineering processes. As a result, the liquid thermal conductivity is worth improving for a better engineering operation. The development of nanofluids is caused by the spread of simple nanoparticles to common liquids, such as glycol, vegetable oil and water, or by the mixing of water with glycol. The nanoparticles are known as metal oxides ($Al_{2} O_{3}$*,* $Fe_{2} O_{3}$*, CuO*), carbon (*CNTs, MWCNT*), nitride, and metal carbide. Numerous researchers looked at different types of nanoparticle mixtures, such as metals (*Al, Cu, Fe*), nanoparticle semiconductors, and metal oxides ($Al_{2} O_{3}$, CuO). There are significant references to nanofluid in the books and articles of Minea^14^, Minkowycz et al.^15^, and Mebarek-Oudina et al.^16^. On the other hand, Fan and Wang^17^, Zhao et al.^18^, Mahian et al.^19,20^, Acharya et al.^21^, Buongiorno et al.^22^, Acharya et al.^23^, and Khan et al.^24^ have written detailed articles on nanofluids.

In the past few years, the production of enhanced heat transfer fluids has earned substantial attention from scientists and scholars. Hybrid nanofluid is called a modern kind of nanofluid which is therefore used to expand the performance of heat transfer. According to Choi and Eastman^25^, “nanofluid is the mixture of solid nanoparticles in the base fluid.” Now, hybrid nanofluid can be explained as a mixture of nanoparticles in the regular nanofluid where the particles of nanofluid should be different. The computational model of Devi and Devi^26^ is more accurate from the various models of the hybrid nanofluid as they have compared their results with the experimental outcomes of Suresh et al.^27^ and obtained in the outstanding agreements. This model is used by numerous scholars such as Yan et al.^28^, Acharya et al.^29^, Lund et al.^30,31^, Waini et al.^32^, Olatundun and Makinde^33^. Waini et al.^34^ examined hybrid nanofluid over the radiated horizontal exponential surface. They examined the two solutions depending on the suction and stretching/shrinking parameter. The same problem was extended by Yan et al.^28^ in which they examined the fluid flow with the effect of the Joule heating and magnetic parameter over the exponential horizontal surface. They have found double solutions depends on the magnetic field.

Many of the hybrid nanofluid flow problems described in the above paragraphs were studied for two-dimensional flow and also did not recognize the impact of multiple solutions for the rotating flow model. In the current examination, we have considered the rotational flow of the hybrid nanofluid of 3D. There are two main objectives of the present study to be considered, one of them is to find the multiple solutions of the model and the second one is to perform the stability analysis of multiple solutions. To the best of the authors' knowledge, such analysis on multiple solutions has not yet been performed in the published literature.

## Mathematical Formulation

**Figure 1:**
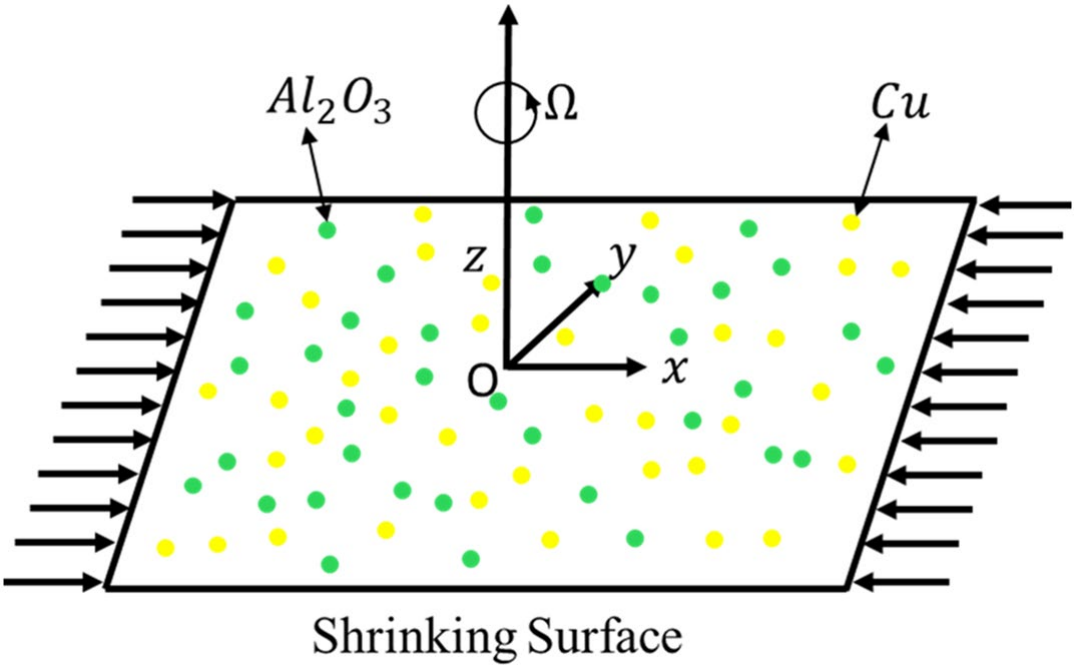
Physical model and coordinate system.

MHD, steady, 3D flow of $Cu - Al_{2} O_{3}$/water hybrid nanofluid along heat transfer on the shrinking surface has been taken into account as revealed in Fig. 1. The sheet at $z = 0$ is velocity in $x$-axis direction i.e., $u_{w} \left( x \right) = - cx$. Mass flux of velocity is $w_{w} \left( x \right) = - S\sqrt {c\vartheta_{f} } ,$ the temperature within (outside) boundary layer is $T_{w}$ ($T_{\infty }$). The porous space is saturated by an incompressible fluid that describes the relationship of Darcy-Forchheimer. Liquid and surface are both rotating with $\Omega_{0}$ constant angular velocity of the z-axis taken to surface as normal. A field of uniform magnetic is positioned in the $z$-axis direction which is $B = B_{0} .$ These consequences in *x*-axis and $z$-axis directions having magnetic effects. As a result, the magnetic field directly effects the $x$-axis and $z$-axis directions. The Reynolds number of magnetic is assumed to be very low, and the field of induced magnetic is disregarded.

Hybrid nanofluid flow equations for temperature and momentum with boundary layer assumptions are expressed as follows^7,9^:

1 $$ u_{x} + v_{y} + w_{z} = 0 $$

2 $$ uu_{x} + vu_{y} + wu_{z} - 2\Omega_{0} v = \frac{{\mu_{hnf} }}{{\rho_{hnf} }}u_{zz} - \frac{1}{{\rho_{hnf} }}\left( {\frac{{\mu_{hnf} }}{{K^{*} }} + \sigma_{hnf} B^{2} } \right)u - Fu^{2} $$

3 $$ uv_{x} + vv_{y} + wv_{z} + 2\Omega_{0} u = \frac{{\mu_{hnf} }}{{\rho_{hnf} }}v_{zz} - \frac{1}{{\rho_{hnf} }}\left( {\frac{{\mu_{hnf} }}{{K^{*} }} + \sigma_{hnf} B^{2} } \right)v - Fv^{2} $$

4 $$ \left( {\rho c_{p} } \right)_{hnf} \left[ {uT_{x} + vT_{y} + wT_{z} } \right] = k_{hnf} T_{zz} - q_{r} $$

The related boundary conditions (BCs) (1–4) are

5 $$ \left\{ {\begin{array}{*{20}l} {v = 0 , u = u_{w} \left( x \right), w = w_{w} \left( x \right), T = T_{w} \quad at\,\, z = 0} \hfill \\ {u \to 0, v \to 0, T \to T_{\infty } , \quad as\,\, z \to \infty } \hfill \\ \end{array} } \right. $$

where $u$, $v$, and $w$ are the corresponding velocity components in $x,y,$ and $z$-axes, $\sigma_{hnf}$ is a hybrid nanofluid electrical conductivity, and $q_{r} = \frac{{ - 4\sigma^{*} }}{{3k^{*} }}\frac{{\partial T^{4} }}{\partial z}$ is the radiative flux where $k^{*}$ and $\sigma^{*}$ are the coefficient of mean absorption and Stefan-Boltzmann constant. Further, $k_{hnf} ,\left( {\rho c_{p} } \right)_{hnf} , \rho_{hnf}$ and $,\mu_{hnf}$ are the corresponding thermal conductivity, heat capacity, density, and dynamic viscosity of hybrid nanofluid. Moreover, $K^{*}$ is the porous medium permeability, $F = \frac{{C_{b} }}{{x\left( {K^{*} } \right)^{1/2} }}$ is the non-uniform inertia coefficient of medium, $C_{b}$ is the drag coefficient. Furthermore, the $hnf$ subscription illustrates the hybrid nanofluid properties. The thermophysical properties are given in Tables 1 and 2.

**Table 1:** Thermophysical properties of hybrid nanofluid^7,9^.

Properties	Hybrid nanofluid
Density	$\rho_{hnf} = \left( {1 - \phi_{2} } \right)\left[ {\left( {1 - \phi_{1} } \right)\rho_{f} + \phi_{1} \rho_{1} } \right] + \phi_{2} \rho_{2}$
Dynamic viscosity	$\mu_{hnf} = \frac{{\mu_{f} }}{{\left( {1 - \phi_{2} } \right)^{2.5} \left( {1 - \phi_{1} } \right)^{2.5} }}$
Thermal conductivity	$k_{hnf} = \frac{{k_{2} + 2k_{nf} - 2\phi_{2} \left( {k_{nf} - k_{2} } \right)}}{{k_{2} + 2k_{nf} + \phi_{2} \left( {k_{nf} - k_{2} } \right)}} \times \left( {k_{nf} } \right)$ where $k_{nf} = \frac{{k_{1} + 2k_{f} - 2\phi_{1} \left( {k_{f} - k_{1} } \right)}}{{k_{1} + 2k_{f} + \phi_{1} \left( {k_{f} - k_{1} } \right)}} \times \left( {k_{f} } \right)$
Electrical conductivity	$\sigma_{hnf} = \frac{{\sigma_{2} + 2\sigma_{nf} - 2\phi_{2} \left( {\sigma_{nf} - \sigma_{2} } \right)}}{{\sigma_{2} + 2\sigma_{nf} + \phi_{2} \left( {\sigma_{nf} - \sigma_{2} } \right)}} \times \left( {\sigma_{nf} } \right)$ where $\sigma_{nf} = \frac{{\sigma_{1} + 2\sigma_{f} - 2\phi_{1} \left( {\sigma_{f} - \sigma_{1} } \right)}}{{\sigma_{1} + 2\sigma_{f} + \phi_{1} \left( {\sigma_{f} - \sigma_{1} } \right)}} \times \left( {\sigma_{f} } \right)$
Heat capacity	$\left( {\rho c_{p} } \right)_{hnf} = \left( {1 - \phi_{2} } \right)\left[ {\left( {1 - \phi_{1} } \right)\left( {\rho c_{p} } \right)_{f} + \phi_{1} \left( {\rho c_{p} } \right)_{1} } \right] + \phi_{2} \left( {\rho c_{p} } \right)_{2}$

**Table 2:** Thermo physical properties of water and particles^7^.

Properties	Water $\left( {H_{2} O} \right)$	Copper (*Cu*)	${\text{Alumina }}\left( {Al_{2} O_{3} } \right)$
$\rho \,\left( {{\text{kg/m}}^{3} } \right)$	997.1	8933	3970
$c_{p} \,\left( {{\text{J/kg}}\,{\text{K}}} \right)$	4179	385	765
$k\, \left( {\text{W/m K}} \right)$	0.613	400	40
$Pr$	6.2		

For this problem, we follow the similarity variables of Anuar et al.^35^

6 $$ u = cxf^{\prime}\left( \eta \right),v = cxg\left( \eta \right), \eta = z\sqrt {\frac{c}{{\vartheta_{f} }}} , w = - \sqrt {c\vartheta_{f} } f\left( \eta \right), \theta \left( \eta \right) = {\raise0.7ex\hbox{${\left( {T - T_{\infty } } \right)}$} \!\mathord{\left/ {\vphantom {{\left( {T - T_{\infty } } \right)} {\left( {T_{w} - T_{\infty } } \right)}}}\right.\kern-\nulldelimiterspace} \!\lower0.7ex\hbox{${\left( {T_{w} - T_{\infty } } \right)}$}} $$

Employing of similarity variable (6) in Eqs. (1–4), one obtains

7 $$ \begin{aligned} & f^{\prime\prime\prime} - Kf^{\prime} + \left\{ {\left( {1 - \phi_{2} } \right)\left[ {1 - \phi_{1} + \phi_{1} \left( {{\raise0.7ex\hbox{${\rho_{1} }$} \!\mathord{\left/ {\vphantom {{\rho_{1} } {\rho_{f} }}}\right.\kern-\nulldelimiterspace} \!\lower0.7ex\hbox{${\rho_{f} }$}}} \right)} \right] + \phi_{2} \left( {{\raise0.7ex\hbox{${\rho_{2} }$} \!\mathord{\left/ {\vphantom {{\rho_{2} } {\rho_{f} }}}\right.\kern-\nulldelimiterspace} \!\lower0.7ex\hbox{${\rho_{f} }$}}} \right)} \right\}\left( {1 - \phi_{2} } \right)^{2.5} \left( {1 - \phi_{1} } \right)^{2.5} \left[ {ff^{\prime\prime} - \left( {1 + F_{s} } \right)f^{^{\prime}2} + 2\Omega g} \right] \\ & \quad - \frac{{\sigma_{hnf} }}{{\sigma_{f} }}\left( {1 - \phi_{2} } \right)^{2.5} \left( {1 - \phi_{1} } \right)^{2.5} Mf^{\prime} = 0 \\ \end{aligned} $$

8 $$ \begin{aligned} & g^{\prime\prime} - Kg + \left\{ {\left( {1 - \phi_{2} } \right)\left[ {1 - \phi_{1} + \phi_{1} \left( {{\raise0.7ex\hbox{${\rho_{1} }$} \!\mathord{\left/ {\vphantom {{\rho_{1} } {\rho_{f} }}}\right.\kern-\nulldelimiterspace} \!\lower0.7ex\hbox{${\rho_{f} }$}}} \right)} \right] + \phi_{2} \left( {{\raise0.7ex\hbox{${\rho_{2} }$} \!\mathord{\left/ {\vphantom {{\rho_{2} } {\rho_{f} }}}\right.\kern-\nulldelimiterspace} \!\lower0.7ex\hbox{${\rho_{f} }$}}} \right)} \right\}\left( {1 - \phi_{2} } \right)^{2.5} \left( {1 - \phi_{1} } \right)^{2.5} \left[ {fg^{\prime} - f^{\prime}g - 2\Omega f^{\prime} - F_{s} g^{2} } \right] \\ & \quad - \frac{{\sigma_{hnf} }}{{\sigma_{f} }}\left( {1 - \phi_{2} } \right)^{2.5} \left( {1 - \phi_{1} } \right)^{2.5} Mg = 0 \\ \end{aligned} $$

9 $$ \frac{1}{{Pr\left\{ {\left( {1 - \phi_{2} } \right)\left[ {1 - \phi_{1} + \phi_{1} \frac{{\left( {\rho c_{p} } \right)_{1} }}{{\left( {\rho c_{p} } \right)_{f} }}} \right] + \phi_{2} \frac{{\left( {\rho c_{p} } \right)_{2} }}{{\left( {\rho c_{p} } \right)_{f} }}} \right\}}}\left( {k_{hnf} /k_{f} + \frac{4}{3}R} \right)\theta^{\prime\prime} + \theta^{\prime}f = 0 $$

Along with BCs

10 $$ \left\{ {\begin{array}{*{20}l} {f\left( 0 \right) = S, f^{\prime}\left( 0 \right) = - 1, g\left( 0 \right) = 0,\theta \left( 0 \right) = 1} \hfill \\ {f^{\prime}\left( \eta \right) \to 0,g\left( \eta \right) \to 0 \theta \left( \eta \right) \to 0, \quad {\text{as}}\,\, \eta \to \infty } \hfill \\ \end{array} } \right. $$

Here prime represents the differentiation of $\eta$, $K = \frac{{\mu_{f} }}{{\rho_{f} cK^{*} }}$ is the parameter of porosity, Prandtl number is $Pr = \frac{{\vartheta_{f} }}{{\alpha_{f} }}$, $\Omega = \frac{{\Omega_{0} }}{c}$ is rotation parameter, inertia coefficient is $F_{s} = \frac{{C_{b} }}{{\sqrt {K^{*} } }}$, $R = \frac{{ - 4\sigma^{*} T_{\infty }^{3} }}{{3k^{*} k_{f} }}$ is the radiation parameter, and $S$ is the suction (injection) parameter when $S > 0$ ($S < 0$).

The coefficient of skin friction and the Nusselt number are two physical quantities of interest that are expressed as follows.

11 $$ \left\{ {\begin{array}{*{20}l} {C_{fx} = \frac{{\mu_{hnf} }}{{\rho_{f} c^{2} }}\left( {\frac{\partial u}{{\partial z}}} \right)\left| {z = 0} \right. } \hfill \\ {C_{fy} = \frac{{\mu_{hnf} }}{{\rho_{f} c^{2} }}\left( {\frac{\partial v}{{\partial z}}} \right)\left| {z = 0} \right. } \hfill \\ {Nu_{x} = - \frac{1}{{k_{f} \left( {T_{w} - T_{\infty } } \right)}}\left[ {k_{hnf} \left( {\frac{\partial T}{{\partial z}}} \right)\left| z \right. + q_{r} \left| z \right.} \right] = 0} \hfill \\ \end{array} } \right. $$

Applying Eq. (6) into Eq. (11) yields to

12 $$ \left\{ {\begin{array}{*{20}l} {\sqrt {Re_{x} } C_{fx} = \frac{1}{{\left( {1 - \phi_{1} } \right)^{2.5} \left( {1 - \phi_{2} } \right)^{2.5} }}f^{\prime\prime}\left( 0 \right)} \hfill \\ {\sqrt {Re_{x} } C_{fy} = \frac{1}{{\left( {1 - \phi_{1} } \right)^{2.5} \left( {1 - \phi_{2} } \right)^{2.5} }}g^{\prime}\left( 0 \right)} \hfill \\ {\sqrt {\frac{1}{{Re_{x} }}} Nu_{x} = - \left( {\frac{{k_{hnf} }}{{k_{f} }} + \frac{4}{3}R} \right)\theta^{\prime}\left( 0 \right)} \hfill \\ \end{array} } \right. $$

where $Re_{x} = cx^{2} /\vartheta_{f}$ is a local Reynold number.

## Temporal stability analysis

The results of Eqs. (7–9) illustrate that there are dual solutions. A stability analysis is then conducted which was done by several scholars in their studies^36,37^. Unsteady equations are supposed to be used for the stability study. Then, Eqs. (2–4) are written as

13 $$ u_{t} + uu_{x} + vu_{y} + wu_{z} - 2\Omega_{0} v = \frac{{\mu_{hnf} }}{{\rho_{hnf} }}u_{zz} - \frac{1}{{\rho_{hnf} }}\left( {\frac{{\mu_{hnf} }}{{K^{*} }} + \sigma_{hnf} B^{2} } \right)u - Fu^{2} $$

14 $$ v_{t} + uv_{x} + vv_{y} + wv_{z} + 2\Omega_{0} u = \frac{{\mu_{hnf} }}{{\rho_{hnf} }}v_{zz} - \frac{1}{{\rho_{hnf} }}\left( {\frac{{\mu_{hnf} }}{{K^{*} }} + \sigma_{hnf} B^{2} } \right)v - Fv^{2} $$

15 $$ \left( {\rho c_{p} } \right)_{hnf} \left[ {T_{t} + uT_{x} + vT_{y} + wT_{z} } \right] = k_{hnf} T_{zz} - q_{r} $$

where $t$ shows the time. Equation (6) can be expressed as follows for unstable flow equations with the new similarity variable $\tau = ct$

16 $$ \left\{ {\begin{array}{*{20}l} { u = cxf_{\eta } \left( {\eta ,\tau } \right),v = cxg\left( {\eta ,\tau } \right), \eta = z\sqrt {\frac{c}{{\vartheta_{f} }}} } \hfill \\ {w = - \sqrt {c\vartheta_{f} } f\left( {\eta ,\tau } \right), \tau = ct} \hfill \\ {\theta \left( {\eta ,\tau } \right) = {\raise0.7ex\hbox{${\left( {T - T_{\infty } } \right)}$} \!\mathord{\left/ {\vphantom {{\left( {T - T_{\infty } } \right)} {\left( {T_{w} - T_{\infty } } \right)}}}\right.\kern-\nulldelimiterspace} \!\lower0.7ex\hbox{${\left( {T_{w} - T_{\infty } } \right)}$}}} \hfill \\ \end{array} } \right. $$

By putting Eq. (16) into Eqs. (13–15) leads to

17 $$ \begin{aligned} & f_{\eta \eta \eta } - Kf_{\eta } + \left\{ {\left( {1 - \phi_{2} } \right)\left[ {1 - \phi_{1} + \phi_{1} \left( {{\raise0.7ex\hbox{${\rho_{1} }$} \!\mathord{\left/ {\vphantom {{\rho_{1} } {\rho_{f} }}}\right.\kern-\nulldelimiterspace} \!\lower0.7ex\hbox{${\rho_{f} }$}}} \right)} \right] + \phi_{2} \left( {{\raise0.7ex\hbox{${\rho_{2} }$} \!\mathord{\left/ {\vphantom {{\rho_{2} } {\rho_{f} }}}\right.\kern-\nulldelimiterspace} \!\lower0.7ex\hbox{${\rho_{f} }$}}} \right)} \right\}\left( {1 - \phi_{2} } \right)^{2.5} \left( {1 - \phi_{1} } \right)^{2.5} \left[ {ff_{\eta \eta } - \left( {1 + F_{s} } \right)\left( {f_{\eta } } \right)^{2} + 2\Omega g - f_{\tau \eta } } \right] \\ & \quad - \frac{{\sigma_{hnf} }}{{\sigma_{f} }}\left( {1 - \phi_{2} } \right)^{2.5} \left( {1 - \phi_{1} } \right)^{2.5} Mf_{\eta } = 0 \\ \end{aligned} $$

18 $$ \begin{aligned} & g_{\eta \eta } - Kg + \left\{ {\left( {1 - \phi_{2} } \right)\left[ {1 - \phi_{1} + \phi_{1} \left( {{\raise0.7ex\hbox{${\rho_{1} }$} \!\mathord{\left/ {\vphantom {{\rho_{1} } {\rho_{f} }}}\right.\kern-\nulldelimiterspace} \!\lower0.7ex\hbox{${\rho_{f} }$}}} \right)} \right] + \phi_{2} \left( {{\raise0.7ex\hbox{${\rho_{2} }$} \!\mathord{\left/ {\vphantom {{\rho_{2} } {\rho_{f} }}}\right.\kern-\nulldelimiterspace} \!\lower0.7ex\hbox{${\rho_{f} }$}}} \right)} \right\}\left( {1 - \phi_{2} } \right)^{2.5} \left( {1 - \phi_{1} } \right)^{2.5} \left[ {fg_{\eta } - f_{\eta } g - 2\Omega f_{\eta } - g_{\tau } } \right] \\ & \quad - \frac{{\sigma_{hnf} }}{{\sigma_{f} }}\left( {1 - \phi_{2} } \right)^{2.5} \left( {1 - \phi_{1} } \right)^{2.5} Mg = 0 \\ \end{aligned} $$

19 $$ \frac{1}{{Pr\left\{ {\left( {1 - \phi_{2} } \right)\left[ {1 - \phi_{1} + \phi_{1} \frac{{\left( {\rho c_{p} } \right)_{1} }}{{\left( {\rho c_{p} } \right)_{f} }}} \right] + \phi_{2} \frac{{\left( {\rho c_{p} } \right)_{2} }}{{\left( {\rho c_{p} } \right)_{f} }}} \right\}}}\left( {k_{hnf} /k_{f} + \frac{4}{3}R} \right)\theta_{\eta \eta } + \theta_{\eta } f - \theta_{\tau } = 0 $$

while BCs (10) becomes:

20 $$ \left\{ {\begin{array}{*{20}l} {f\left( {0,\tau } \right) = S, f_{\eta } \left( {0,\tau } \right) = - 1, g\left( {0,\tau } \right) = 0,\theta \left( {0,\tau } \right) = 1} \hfill \\ {f_{\eta } \left( {\eta ,\tau } \right) \to 0,g\left( {\eta ,\tau } \right) \to 0 \theta \left( {\eta ,\tau } \right) \to 0, \quad {\text{as}}\,\, \eta \to \infty } \hfill \\ \end{array} } \right. $$

As suggested by Weidman et al.^38^, “the stability of the steady flow solutions $f\left( \eta \right) = f_{0} \left( \eta \right),g\left( \eta \right) = g_{0} \left( \eta \right)$ and $\theta \left( \eta \right) = \theta_{0} \left( \eta \right)$ are identified by writing $F\left( {\eta ,\tau } \right), G\left( {\eta ,\tau } \right)$ and $H\left( {\eta ,\tau } \right)$” as follows

21 $$ f\left( {\eta ,\tau } \right) = f_{0} \left( \eta \right) + e^{ - \varepsilon \tau } F\left( {\eta ,\tau } \right), g\left( {\eta ,\tau } \right) = g_{0} \left( \eta \right) + e^{ - \varepsilon \tau } G\left( {\eta ,\tau } \right), \theta \left( {\eta ,\tau } \right) = \theta_{0} \left( \eta \right) + e^{ - \varepsilon \tau } H\left( {\eta ,\tau } \right) $$

where $\varepsilon$ is the unknown eigenvalue parameter, while functions $F\left( {\eta ,\tau } \right), G\left( {\eta ,\tau } \right)$ and $H\left( {\eta ,\tau } \right)$ are perturbated functions to $f\left( \eta \right) = f_{0} \left( \eta \right),g\left( \eta \right) = g_{0} \left( \eta \right)$ and $\theta \left( \eta \right) = \theta_{0} \left( \eta \right)$. By putting the Eq. (21) into Eqs. (17–19), the following system of equations is gotten.

22 $$ \begin{aligned} & F_{\eta \eta \eta } - KF_{\eta } + \left\{ {\left( {1 - \phi_{2} } \right)\left[ {1 - \phi_{1} + \phi_{1} \left( {{\raise0.7ex\hbox{${\rho_{1} }$} \!\mathord{\left/ {\vphantom {{\rho_{1} } {\rho_{f} }}}\right.\kern-\nulldelimiterspace} \!\lower0.7ex\hbox{${\rho_{f} }$}}} \right)} \right] + \phi_{2} \left( {{\raise0.7ex\hbox{${\rho_{2} }$} \!\mathord{\left/ {\vphantom {{\rho_{2} } {\rho_{f} }}}\right.\kern-\nulldelimiterspace} \!\lower0.7ex\hbox{${\rho_{f} }$}}} \right)} \right\}\left( {1 - \phi_{2} } \right)^{2.5} \left( {1 - \phi_{1} } \right)^{2.5} \left[ {f_{0} F_{\eta \eta } + F\left( {f_{0} } \right)_{\eta \eta } - 2\left( {1 + F_{s} } \right)\left( {f_{0} } \right)_{\eta } F_{\eta } + 2\Omega G + \varepsilon F_{\eta } } \right] \\ & \quad - \frac{{\sigma_{hnf} }}{{\sigma_{f} }}\left( {1 - \phi_{2} } \right)^{2.5} \left( {1 - \phi_{1} } \right)^{2.5} MF_{\eta } = 0 \\ \end{aligned} $$

23 $$ \begin{aligned} & G_{\eta \eta } - KG + \left\{ {\left( {1 - \phi_{2} } \right)\left[ {1 - \phi_{1} + \phi_{1} \left( {{\raise0.7ex\hbox{${\rho_{1} }$} \!\mathord{\left/ {\vphantom {{\rho_{1} } {\rho_{f} }}}\right.\kern-\nulldelimiterspace} \!\lower0.7ex\hbox{${\rho_{f} }$}}} \right)} \right] + \phi_{2} \left( {{\raise0.7ex\hbox{${\rho_{2} }$} \!\mathord{\left/ {\vphantom {{\rho_{2} } {\rho_{f} }}}\right.\kern-\nulldelimiterspace} \!\lower0.7ex\hbox{${\rho_{f} }$}}} \right)} \right\}\left( {1 - \phi_{2} } \right)^{2.5} \left( {1 - \phi_{1} } \right)^{2.5} \left[ {f_{0} G_{\eta } + F\left( {g_{0} } \right)_{\eta } - \left( {f_{0} } \right)_{\eta } G - F_{\eta } g_{0} - 2\Omega F_{\eta } + \varepsilon G} \right] \\ & \quad - \frac{{\sigma_{hnf} }}{{\sigma_{f} }}\left( {1 - \phi_{2} } \right)^{2.5} \left( {1 - \phi_{1} } \right)^{2.5} MG = 0 \\ \end{aligned} $$

24 $$ \frac{1}{{Pr\left\{ {\left( {1 - \phi_{2} } \right)\left[ {1 - \phi_{1} + \phi_{1} \frac{{\left( {\rho c_{p} } \right)_{1} }}{{\left( {\rho c_{p} } \right)_{f} }}} \right] + \phi_{2} \frac{{\left( {\rho c_{p} } \right)_{2} }}{{\left( {\rho c_{p} } \right)_{f} }}} \right\}}}\left( {k_{hnf} /k_{f} + \frac{4}{3}R} \right)H_{\eta \eta } + f_{0} H_{\eta } + F\left( {\theta_{0} } \right)_{\eta } + \varepsilon H = 0{ } $$

The BCs are as follows:

25 $$ \left\{ {\begin{array}{*{20}c} {F\left( {0,\tau } \right) = 0, F_{\eta } \left( {0,\tau } \right) = 0, G\left( {0,\tau } \right) = 0,H\left( {0,\tau } \right) = 0} \\ {F_{\eta } \left( {\eta ,\tau } \right) \to 0,G\left( {\eta ,\tau } \right) \to 0, H\left( {\eta ,\tau } \right) \to 0, as \eta \to \infty } \\ \end{array} } \right. $$

The solutions stability of steady flow and heat transfer $f_{0} \left( \eta \right), g_{0} \left( \eta \right)$ and $\theta_{0} \left( \eta \right)$ can be gotten by using $\tau \to 0$. Therefore, functions $F\left( {\eta ,\tau } \right) = F_{0} \left( \eta \right)$, $G\left( {\eta ,\tau } \right) = G_{0} \left( \eta \right)$, and $H\left( {\eta ,\tau } \right) = H_{0} \left( \eta \right)$ are expressed in Eqs. (22–25). As a result, the following is a linearized eigenvalue problem system:

26 $$ \begin{aligned} & F_{0}^{\prime \prime \prime } - KF_{0}^{^{\prime}} + \left\{ {\left( {1 - \phi_{2} } \right)\left[ {1 - \phi_{1} + \phi_{1} \left( {{\raise0.7ex\hbox{${\rho_{1} }$} \!\mathord{\left/ {\vphantom {{\rho_{1} } {\rho_{f} }}}\right.\kern-\nulldelimiterspace} \!\lower0.7ex\hbox{${\rho_{f} }$}}} \right)} \right] + \phi_{2} \left( {{\raise0.7ex\hbox{${\rho_{2} }$} \!\mathord{\left/ {\vphantom {{\rho_{2} } {\rho_{f} }}}\right.\kern-\nulldelimiterspace} \!\lower0.7ex\hbox{${\rho_{f} }$}}} \right)} \right\}\left( {1 - \phi_{2} } \right)^{2.5} \left( {1 - \phi_{1} } \right)^{2.5} \left\{ {f_{0} F_{0}^{^{\prime\prime}} + F_{0} f_{0}^{^{\prime\prime}} - 2\left( {1 + F_{s} } \right)f_{0}^{^{\prime}} F_{0}^{^{\prime}} + \varepsilon F_{0}^{^{\prime}} + 2\Omega G_{0} } \right\} \\ & \quad - \frac{{\sigma_{hnf} }}{{\sigma_{f} }}\left( {1 - \phi_{2} } \right)^{2.5} \left( {1 - \phi_{1} } \right)^{2.5} MF_{0}^{^{\prime}} = 0 \\ \end{aligned} $$

27 $$ \begin{aligned} & G_{0}^{^{\prime\prime}} - KG_{0} + \left\{ {\left( {1 - \phi_{2} } \right)\left[ {1 - \phi_{1} + \phi_{1} \left( {{\raise0.7ex\hbox{${\rho_{1} }$} \!\mathord{\left/ {\vphantom {{\rho_{1} } {\rho_{f} }}}\right.\kern-\nulldelimiterspace} \!\lower0.7ex\hbox{${\rho_{f} }$}}} \right)} \right] + \phi_{2} \left( {{\raise0.7ex\hbox{${\rho_{2} }$} \!\mathord{\left/ {\vphantom {{\rho_{2} } {\rho_{f} }}}\right.\kern-\nulldelimiterspace} \!\lower0.7ex\hbox{${\rho_{f} }$}}} \right)} \right\}\left( {1 - \phi_{2} } \right)^{2.5} \left( {1 - \phi_{1} } \right)^{2.5} \left[ {G_{0}^{^{\prime}} f_{0} + g_{0}^{^{\prime}} F_{0} - \left( {f_{0}^{^{\prime}} G_{0} + F_{0}^{^{\prime}} g_{0} } \right) - 2\Omega F_{0}^{^{\prime}} + \varepsilon G_{0} } \right] \\ & \quad - \frac{{\sigma_{hnf} }}{{\sigma_{f} }}\left( {1 - \phi_{2} } \right)^{2.5} \left( {1 - \phi_{1} } \right)^{2.5} G_{0} = 0 \\ \end{aligned} $$

28 $$ \frac{1}{{Pr\left\{ {\left( {1 - \phi_{2} } \right)\left[ {1 - \phi_{1} + \phi_{1} \frac{{\left( {\rho c_{p} } \right)_{1} }}{{\left( {\rho c_{p} } \right)_{f} }}} \right] + \phi_{2} \frac{{\left( {\rho c_{p} } \right)_{2} }}{{\left( {\rho c_{p} } \right)_{f} }}} \right\}}}\left( {k_{hnf} /k_{f} + \frac{4}{3}R} \right)H_{0}^{^{\prime\prime}} + \theta_{0}^{^{\prime}} F_{0} + H_{0}^{^{\prime}} f_{0} + \varepsilon H_{0} = 0 $$

subject to the following BCs:

29 $$ \left\{ {\begin{array}{*{20}l} {F_{0} \left( 0 \right) = 0, F_{0}^{^{\prime}} \left( 0 \right) = 0, G_{0} \left( 0 \right) = 0,H_{0} \left( 0 \right) = 0} \hfill \\ {F_{0}^{^{\prime}} \left( \eta \right) \to 0,G_{0} \left( \eta \right) \to 0 H_{0} \left( \eta \right) \to 0,\quad {\text{ as}}\,\, \eta \to \infty } \hfill \\ \end{array} } \right. $$

It should be noted that values of the smallest eigenvalue ($\varepsilon$) are found by relaxing boundary conditions^39^. In this problem, we have relaxed $F_{0}^{^{\prime}} \left( \eta \right) \to 0$ as $\eta \to \infty$ into $F_{0}^{^{\prime\prime}} \left( 0 \right) = 1$ as suggested by Harris et al.^39^.

## Results and discussion

The system of ODEs (7–9) subject to BCs (10) has been numerically solved with the Maple computational software with the aid of shootlib function. We have compared the values of $\sqrt{Re}{C}_{fx}$ and $\sqrt{Re}{C}_{fy}$ with Zaimi et al.^40^’s results for pure water (i.e., ${\phi }_{1}={\phi }_{2}=$ 0) over the stretching surface (i.e., ${f}^{\boldsymbol{^{\prime}}}\left(0\right)=1$) in Table 3. It is concluded from the results of Table 3 that the findings indicate excellent agreement with the previous study. It can also be concluded that the present code can be used confidently to investigate the problem under consideration in current work.

**Table 3:** Comparison of values of $\sqrt {Re} C_{fx}$ and $\sqrt {Re} C_{fy}$ for different values of ${\Omega }$ when $f^{\prime}\left( 0 \right) = 1,\phi_{1} = \phi_{2} = S = F_{s} = K = 0$.

${\Omega }$	$\sqrt {Re} C_{fx}$		$\sqrt {Re} C_{fy}$	
	^40^	Present results	^40^	Present results
0	− 1.0000	− 1.0000625	0.0000	0.0000000
0.5	− 1.1384	− 1.1383806	− 0.5128	− 0.5127602
1	− 1.3250	− 1.3250287	− 0.8371	− 0.8370983
2	− 1.6523	− 1.6523520	− 1.2873	− 1.2872588
3	− 1.9289	− 1.9289315	− 1.6248	− 1.6247357
4	− 2.1716	− 2.1715931	− 1.9054	− 1.9053929
5	− 2.3901	− 2.3901398	− 2.1506	− 2.1505265

Making the required hybrid nanofluid, which is $Cu-A{l}_{2}{O}_{3}$*/*water, we mixed the alumina firstly in water, and then copper in the $A{l}_{2}{O}_{3}$*/*water nanofluid. So, ${\phi }_{1}$ is used for the $A{l}_{2}{O}_{3}$ particles and ${\phi }_{2}$ is used for $Cu$ particles. The effect of various physical parameters such as coefficient of inertia ($1\le {F}_{s}<2.4$), rotation parameter ($\Omega \le 0.02$), porosity parameter $(0.4\le K\le 1)$, the volume fraction of particles (${\phi }_{1}=0.1, 0\le {\phi }_{2}\le$ 0.05), magnetic number $(0.4\le M\le 1),$ suction parameter ($S>1.7$), and thermal radiation parameter $(0\le R\le 1)$ are conversed and illustrated in figures.

**Figure 2:**
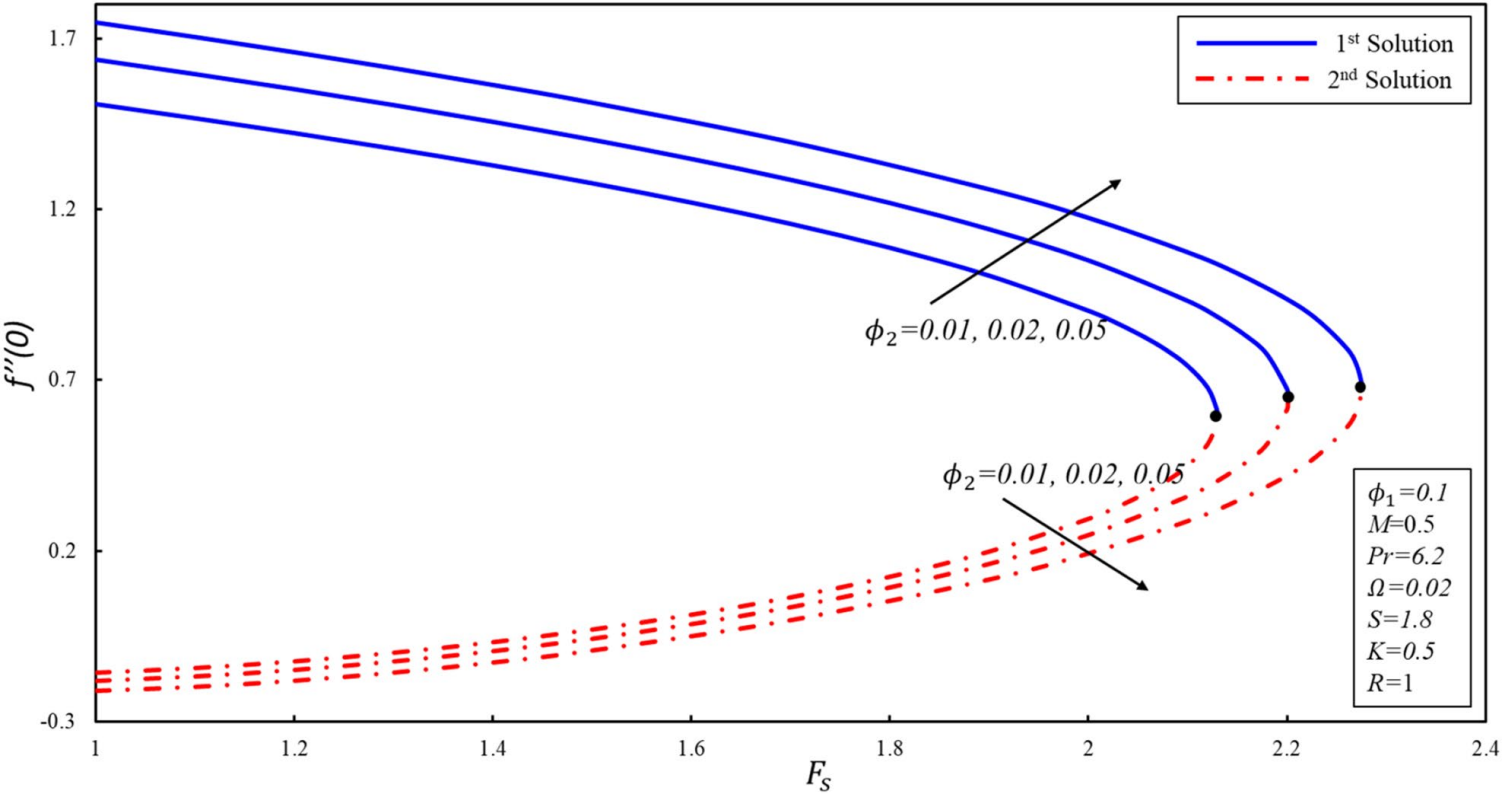
Variation of $f^{\prime \prime}(0)$ for ${\mathrm{\varnothing }}_{2}$ against ${F}_{s}$.

**Figure 3:**
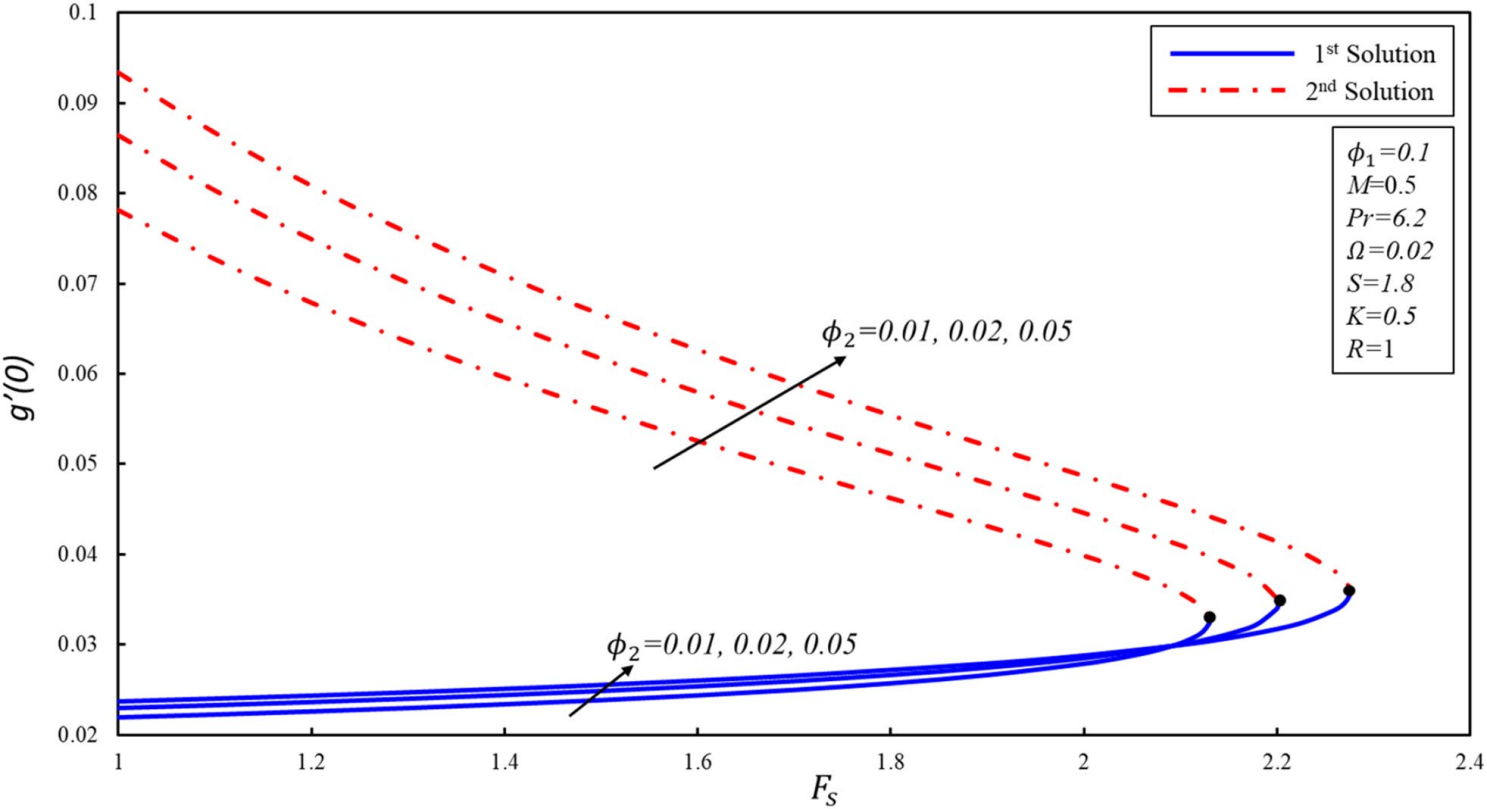
Variation of $g^{\prime}(0)$ for ${\mathrm{\varnothing }}_{2}$ against ${F}_{s}$.

**Figure 4:**
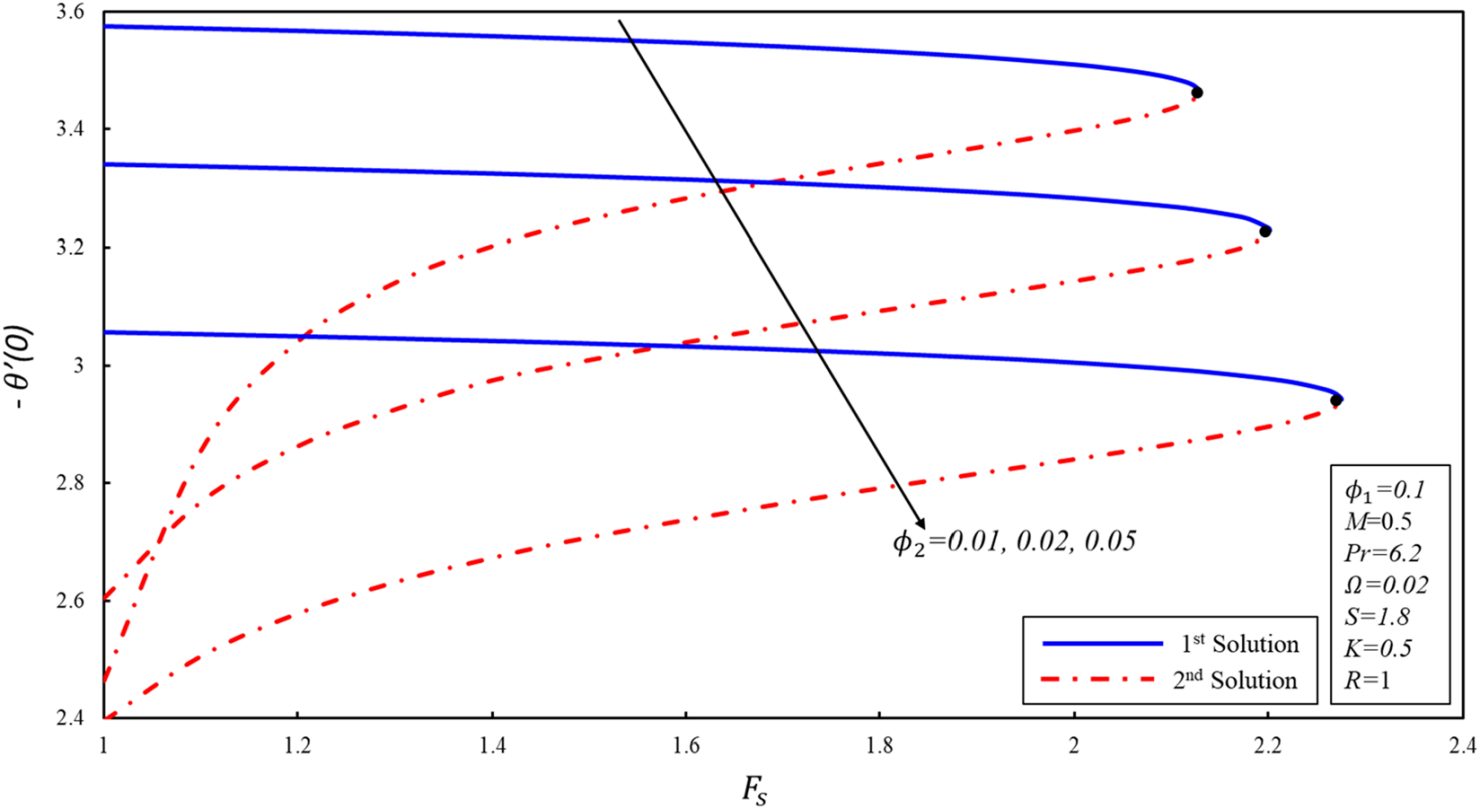
Variation of $-\theta ^{\prime}(0)$ for ${\mathrm{\varnothing }}_{2}$ against ${F}_{s}$.

The presence of multiple solutions enables us to investigate the limits of the parameters where these solutions are possible. Figures 2, 3 and 4 show variants of reduced skin friction coefficient $f^{\prime\prime}\left( 0 \right),g^{\prime}\left( 0 \right),$ and heat transfer rate $- \theta^{\prime } \left( 0 \right)$ for specific values of $\phi_{2}$. The corresponding critical values of $\phi_{2} = 0.01, 0.05, 0.1$ are $F_{sc} = 2.1296, 2.2012, 2.2753$, where $F_{sc}$ is the critical point where both solutions exist at $F_{s} = F_{sc}$. Dual solutions are observed as $F_{s} \le F_{sc}$ and no solution exists when $F_{s} > F_{sc}$. The estimation of boundary layers beyond such critical values is no longer justified. Reduced skin friction coefficient ($f^{\prime\prime}\left( 0 \right)$) increases when $\emptyset_{2}$ is increased in the first solution, physically it is due to the fact that higher values of volume fraction help to reduce the boundary layer thickness, while it reduces in the second solution. Further, the magnitude of $g^{\prime } \left( 0 \right)$ increases when $\emptyset_{2}$ increases in both solutions. Reduced heat transfer rate ($- \theta^{\prime } \left( 0 \right)$) enhances in the second solution when $F_{s}$ increases, while a reverse trend is observed in the stable solution. The enhancement in the magnitude of $- \theta^{\prime } \left( 0 \right)$ is due to the high fraction created by the inertia coefficient.

**Figure 5:**
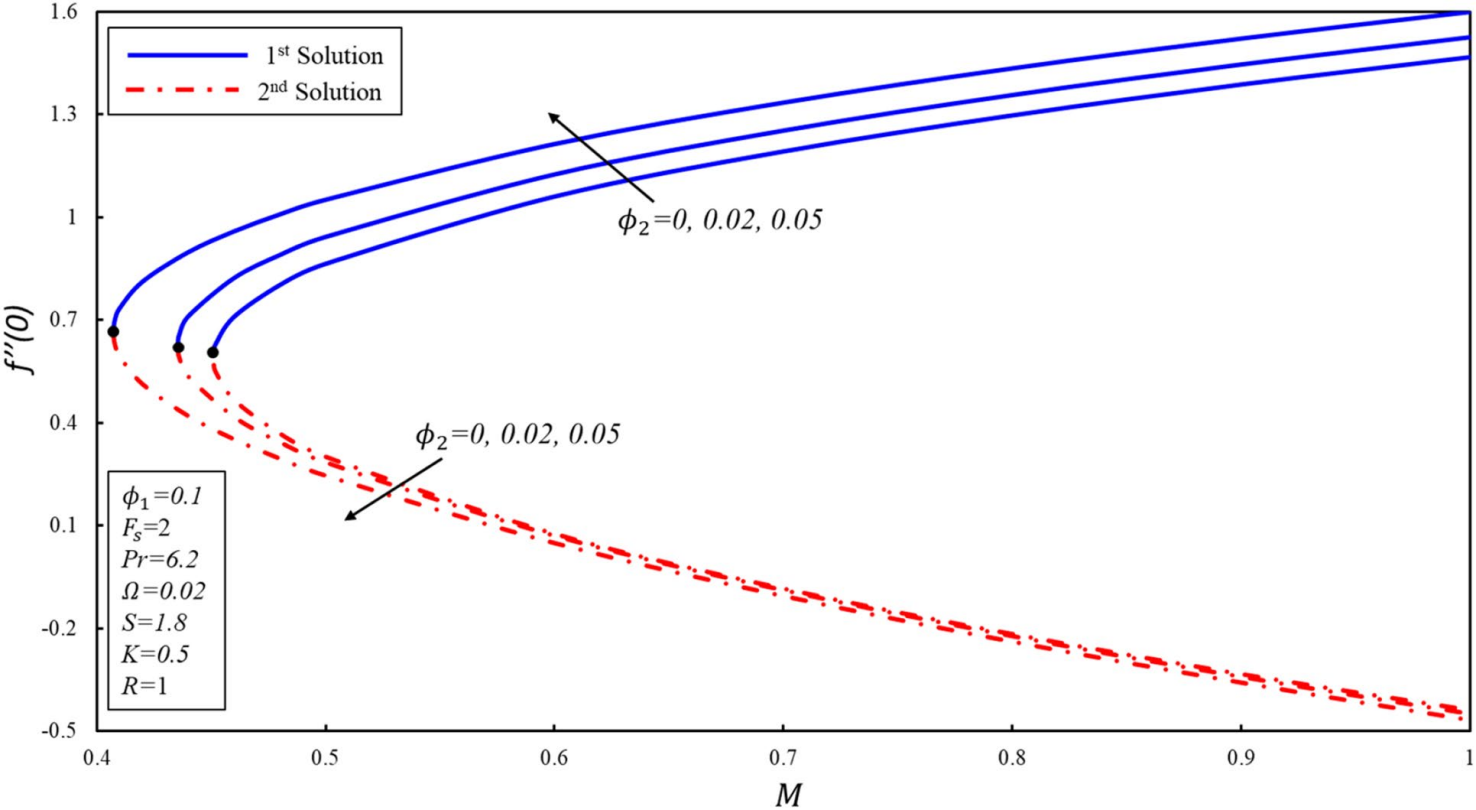
Variation of ${f}^{\prime \prime}\left(0\right)$ for ${\mathrm{\varnothing }}_{2}$ against $M$*.*

**Figure 6:**
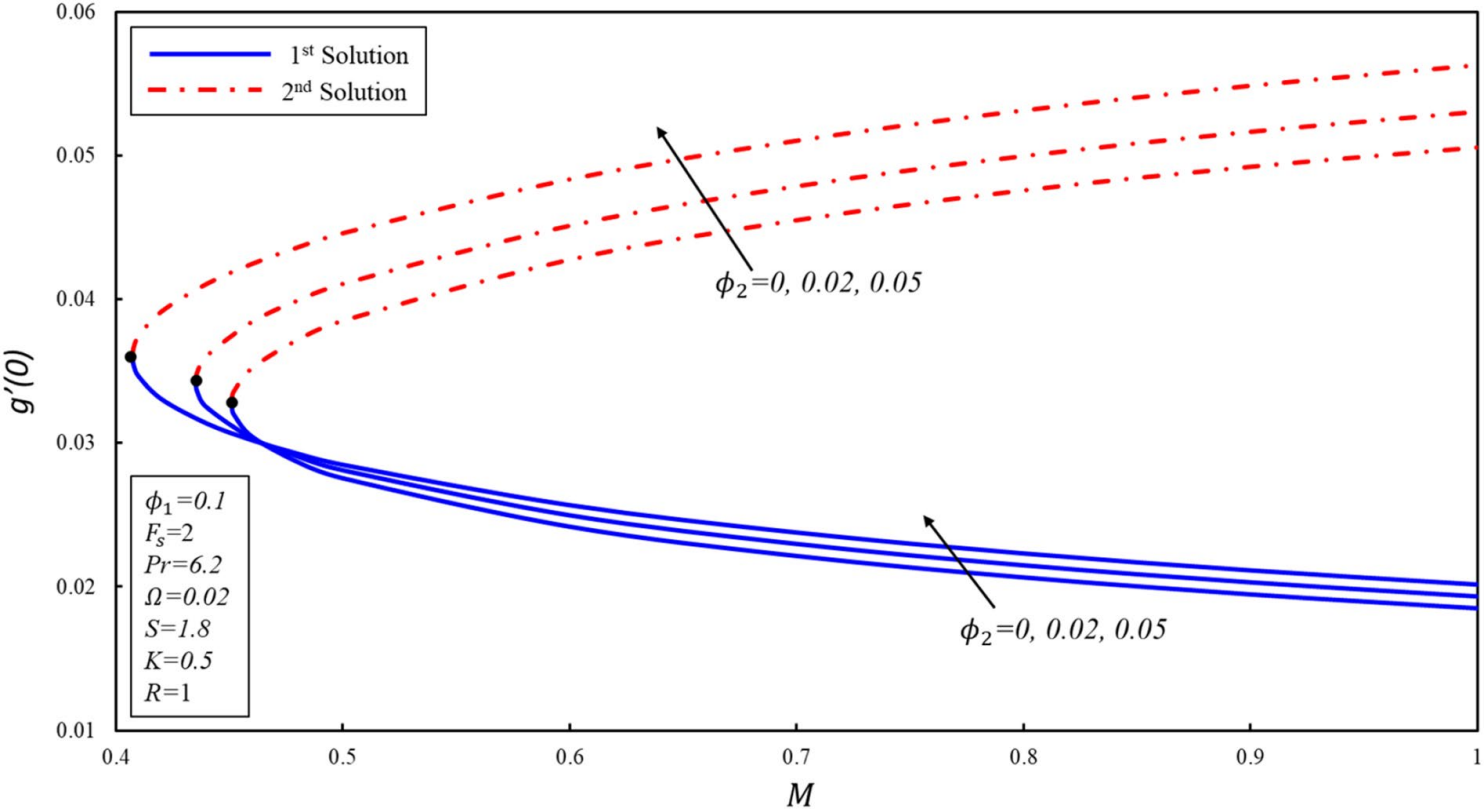
Variation of $g^{\prime}\left( 0 \right)$ for $\emptyset_{2}$ against $M$*.*

**Figure 7:**
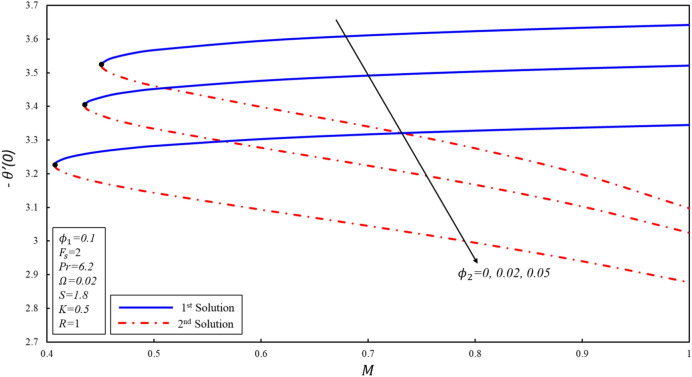
Variation of $- \theta ^{\prime}\left( 0 \right)$ for $\emptyset_{2}$ against $M$*.*

Figures 5, 6 and 7 display the $f^{\prime \prime}(0),g^{\prime}(0)$ and $-\theta ^{\prime}(0)$ variations for various values of magnetic parameter. The critical values of ${\phi }_{2}=0, 0.02, 0.05$ are ${M}_{c}=\mathrm{0.4513,0.4353}, 0.4073$ respectively, where ${M}_{c}$ is critical point where both solutions exist. Dual solutions are observed as $M\ge {M}_{C}$ and no solution exists when $M<{M}_{c}$. The estimation of boundary layers beyond such critical values is no longer justified. Reduced skin friction coefficient ($f^{\prime \prime}(0)$) increases when ${\varnothing }_{2}$ is increased in the first solution, while it reduces in the second solution. Moreover, $f^{\prime \prime}(0)$ increases in the first solution when $M$ increases by keeping a fixed value of ${\varnothing }_{2}$, while the reverse trend is noticed in the second solution. Physically, this phenomenon is attributed to the reason that the Lorenz force repressed the vortex generated by shrinking surface within the boundary layer. Further, $g^{\prime}(0)$ increases when ${\varnothing }_{2}$ increases in both solutions. Physically, the higher volume fraction creates more resistance during the flow of hybrid nanofluid within the boundary layer, resulting in an increase in $g^{\prime}(0)$. Reduced heat transfer ($-\theta ^{\prime}(0)$) increases for the first solution when the magnitude of $M$ enhances, while a reverse trend is detected for the second solution. It should be noted that modeled the equations reveal the alumina model of nanofluid when ${\varnothing }_{2}=0.$

**Figure 8:**
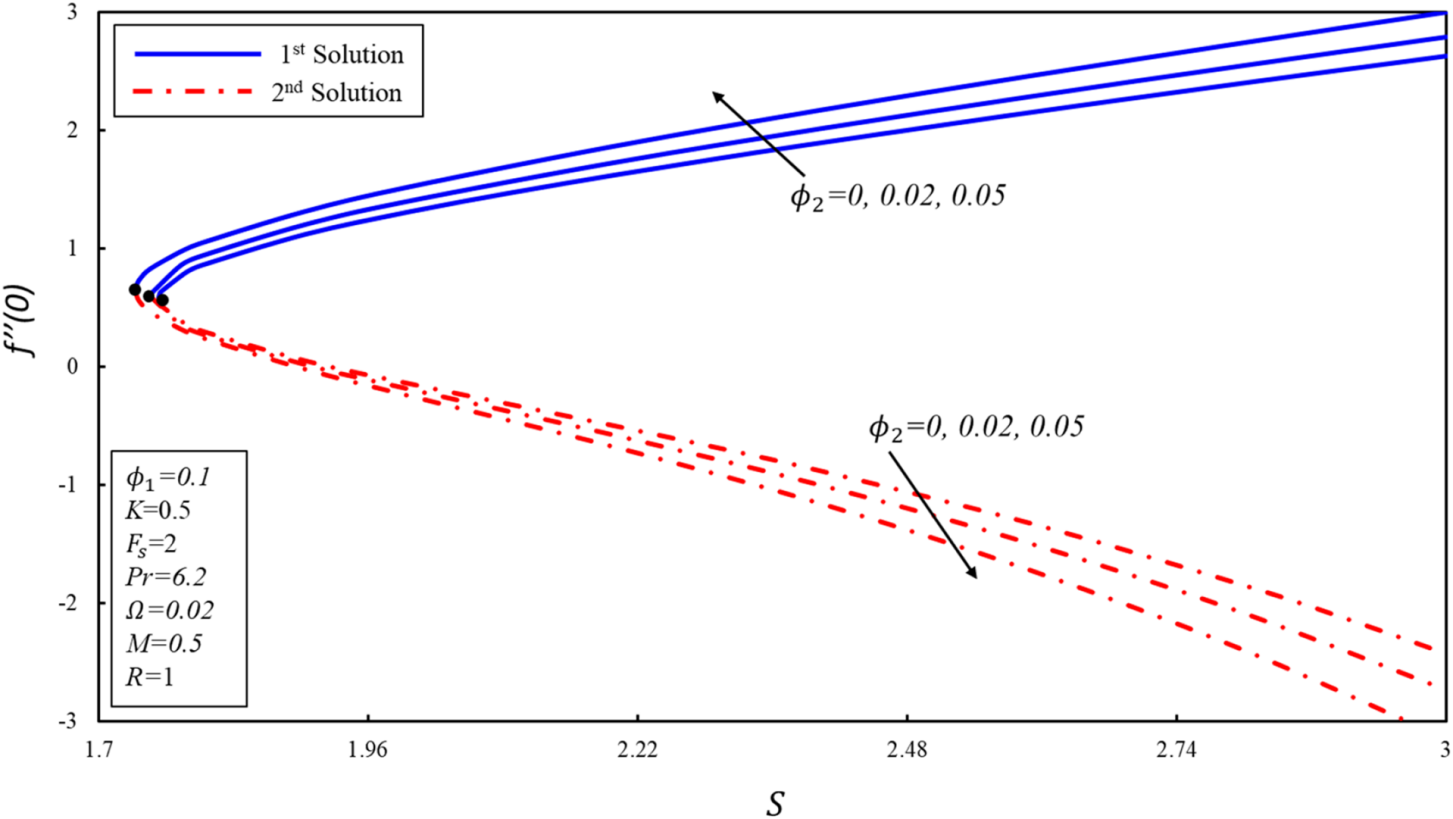
Variation of $f^{\prime\prime}\left( 0 \right)$ for $\emptyset_{2}$ against $S$.

**Figure 9:**
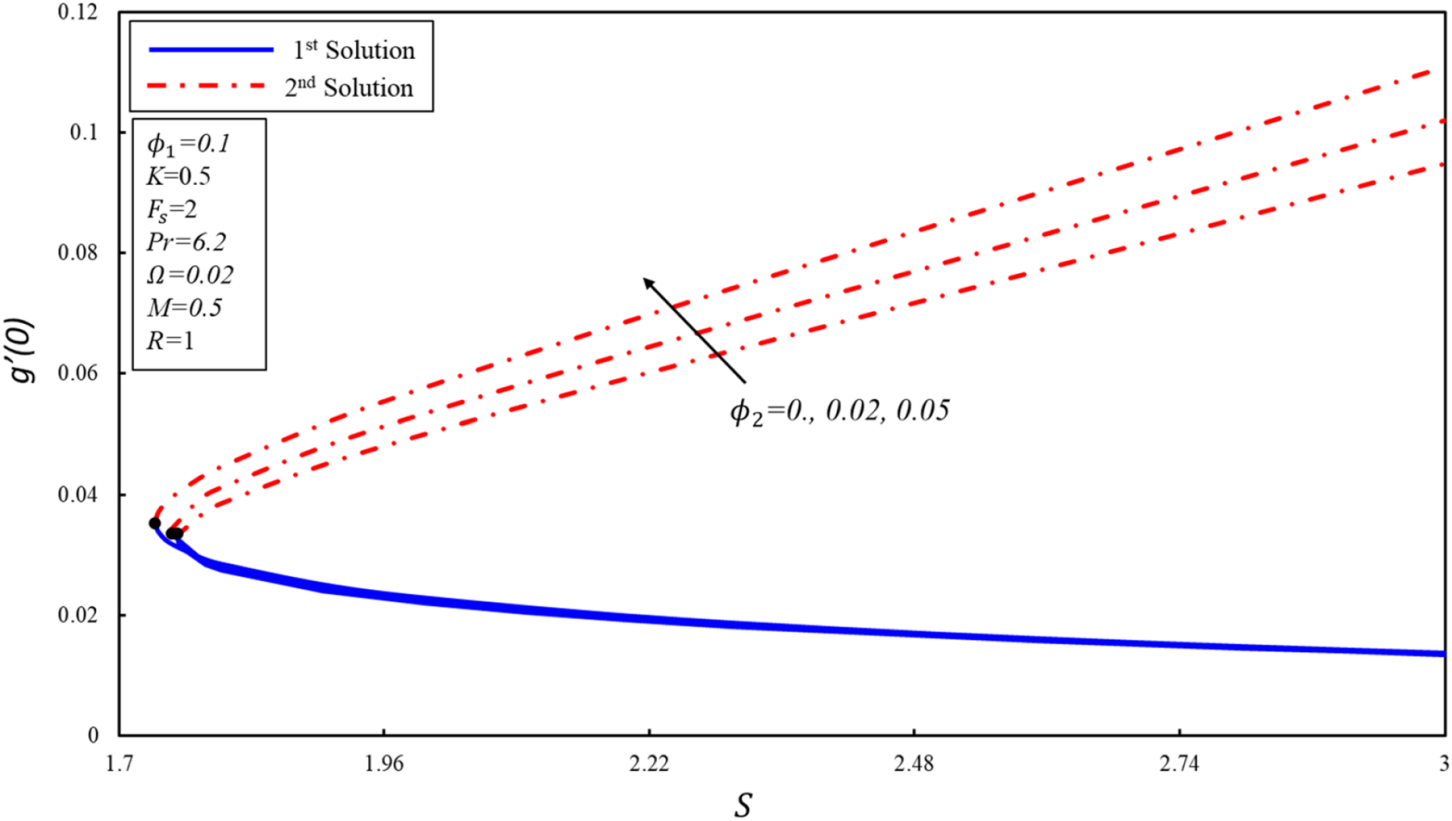
Variation of $g^{\prime}\left( 0 \right)$ for $\emptyset_{2}$ against $S$.

**Figure 10:**
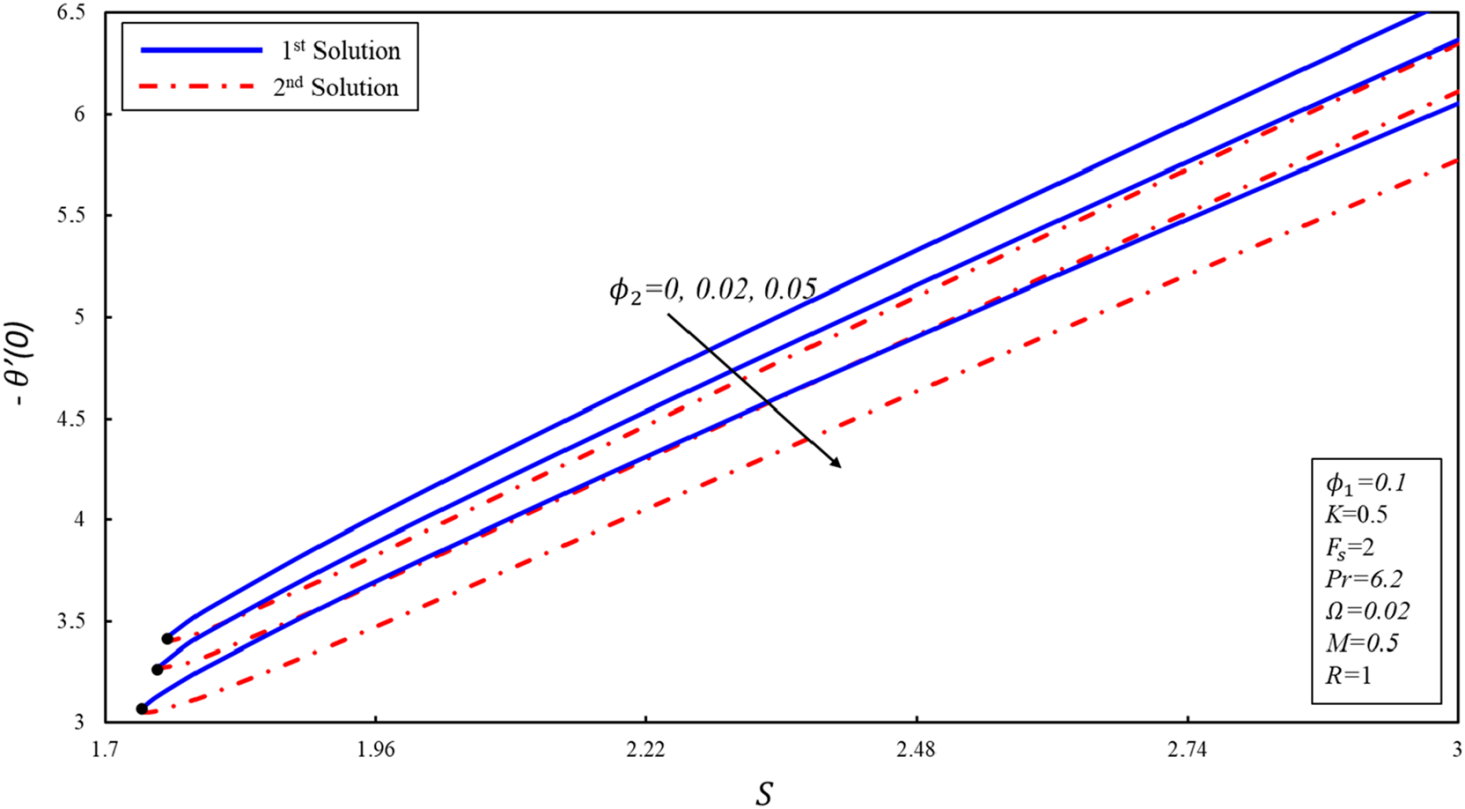
Variation of $- \theta ^{\prime}\left( 0 \right)$ for $\emptyset_{2}$ against $S$.

The variation of $f^{\prime \prime}(0),g^{\prime}(0)$ and $-\theta ^{\prime}(0)$ with suction $S$ for numerous values of solid volume fraction of the copper (${\phi }_{2}$) are presented in Figures 8, 9 and 10. The critical values of ${\phi }_{2}=0, 0.02, 0.05$ are ${S}_{c}=\mathrm{1.7582,1.75}, 1.7364$ respectively, where ${S}_{c}$ is critical point where both solutions exist. Dual solutions are observed when $S\ge {S}_{C}$ and no solution exists when $S<{S}_{c}$. It is worth noting that no solution exists outside of critical values of $S$ (i.e., ${S}_{c}$). Reduced skin friction coefficient ($f^{\prime \prime}(0)$) increases when ${\varnothing }_{2}$ and $S$ are increased in the first solution, physically, this is due to higher copper volume fraction values aiding in the reduction of boundary layer thickness, while it reduces in the second solution as both applied parameters increase. Further, $g^{\prime}(0)$ increases when ${\varnothing }_{2}$ increases in the second solution, while no change is noticed in the behavior of $g^{\prime}(0)$ in the first solution. Moreover, $g^{\prime}(0)$ increases in the second solution when $S$ increases by keeping a fixed value of ${\varnothing }_{2}$, the physical reason for this increase is that the suction creates less resistance in fluid flow. On the other hand, the reverse trend is noticed in the first solution. Reduced heat transfer rate ($-\theta ^{\prime}(0)$) enhances in both solutions when $S$ increases, while reverse trend is observed when ${\varnothing }_{2}$ increases. It is then inferred that the importance of suction is essential in deciding the presence of non-unique solutions.

**Figure 11:**
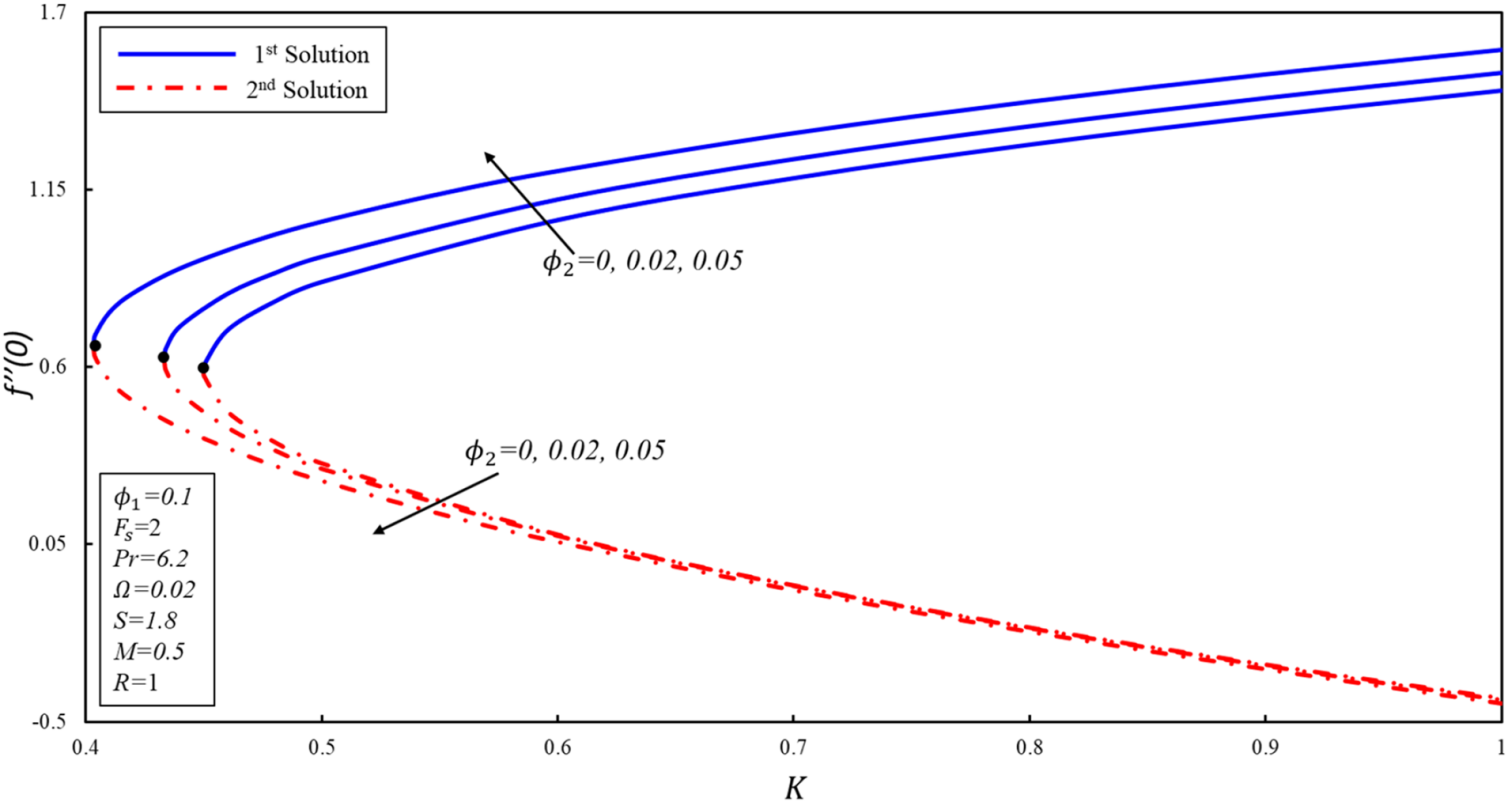
Variation of $f^{\prime\prime}\left( 0 \right)$ for $\emptyset_{2}$ against $K$.

**Figure 12:**
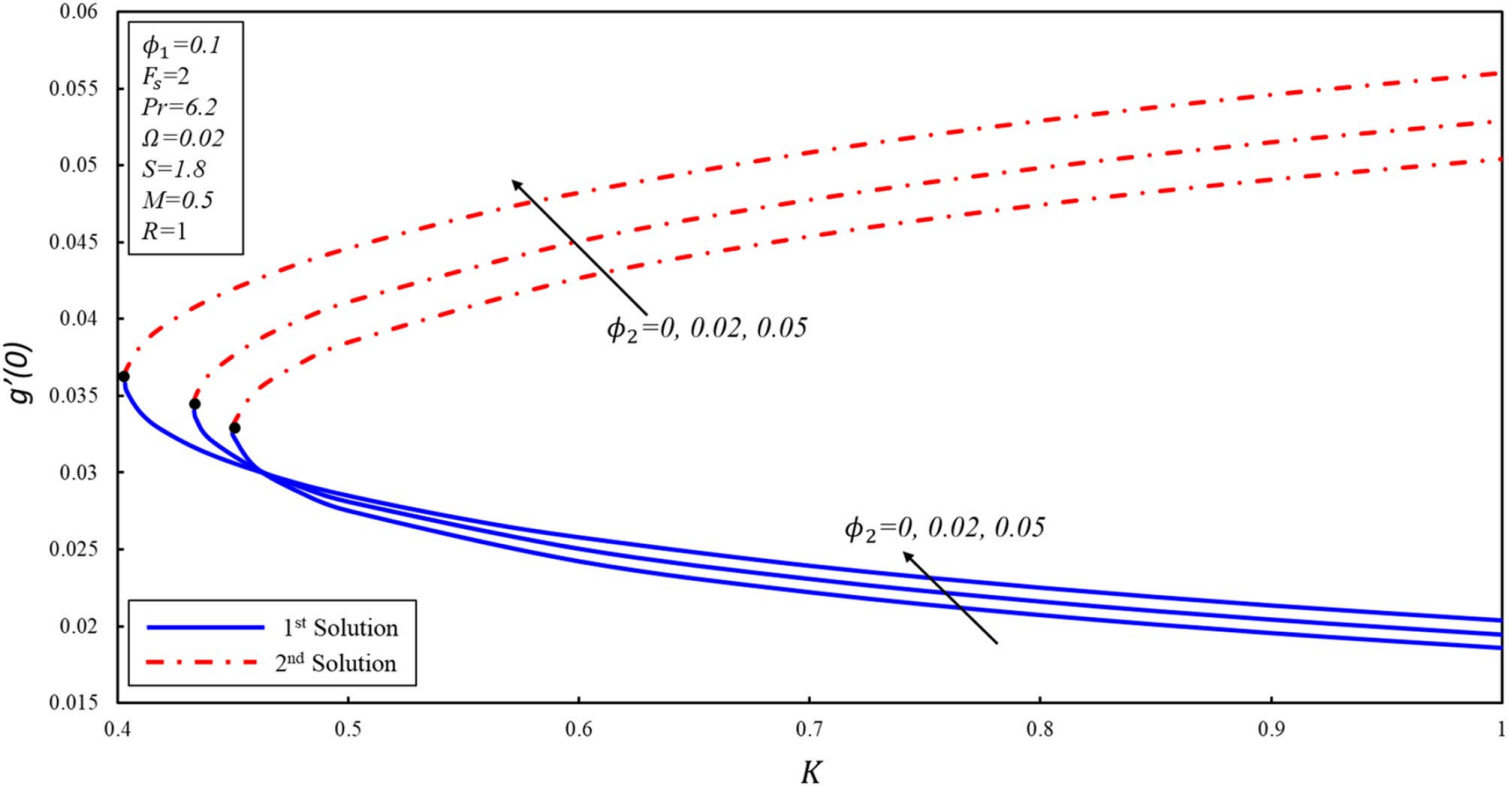
Variation of $g^{\prime}\left( 0 \right)$ for $\emptyset_{2}$ against $K$.

**Figure 13:**
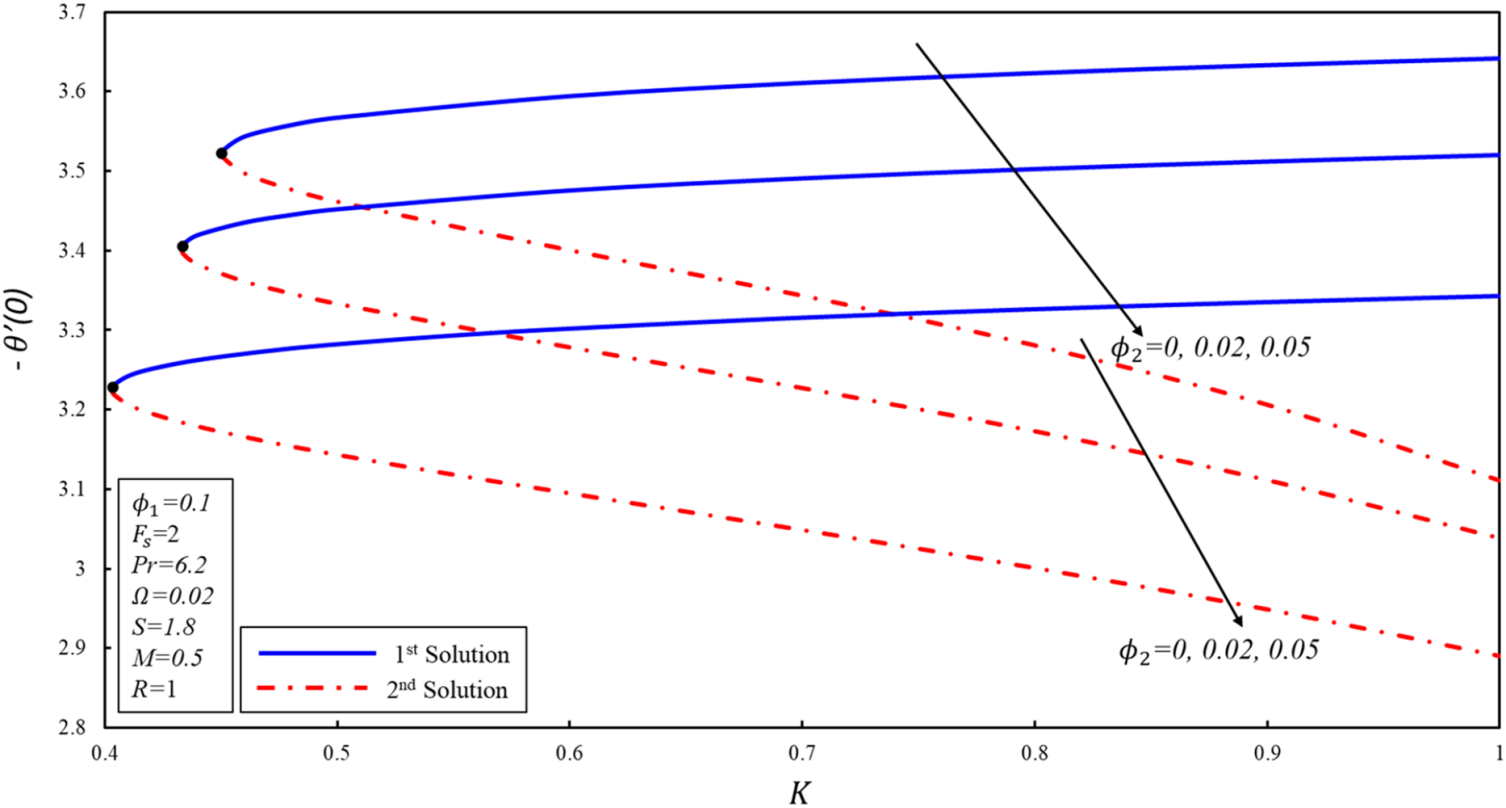
Variation of $- \theta ^{\prime}\left( 0 \right)$ for $\emptyset_{2}$ against $K$.

The effect of porosity parameter $K$ on the magnitude of ${f}^{\prime \prime}\left(0\right),{g}^{^{\prime}}\left(0\right),$ and $-\theta ^{\prime}(0)$ for numerous values of ${\phi }_{2}$ have been plotted in Figures 11, 12 and 13. The corresponding critical values of ${\phi }_{2}=0, 0.02, 0.05$ are ${K}_{c}=\mathrm{0.4499,0.4332}, 0.4033$, where ${K}_{c}$ is the critical point where both solutions exist. Dual solutions are observed when ${K}_{C}\le K$ and no solution exists when ${K}_{C}>K$. It is worth mentioning that beyond ${K}_{C}$ values, no solution exists. Reduced skin friction ($f^{\prime \prime}(0)$) increases when ${\varnothing }_{2}$ and $K$ increase in stable solution, while it reduces in the second solution as both applied parameters increase. Further, $g^{\prime}(0)$ increases when ${\varnothing }_{2}$ increases in both solutions. Moreover, $g^{\prime}(0)$ rises (declines) for the first (second) solution when $K$ increases by keeping a fixed value of ${\varnothing }_{2}$. Reduced heat transfer ($-\theta ^{\prime}(0)$) reduces in both solutions when ${\varnothing }_{2}$ increases, while a reverse trend is observed when $K$ increases in the first solution by keeping the constant values ${\varnothing }_{2}$. This is therefore determined that the porosity values are significant in evaluating the occurrence of non-unique solutions.

**Figure 14:**
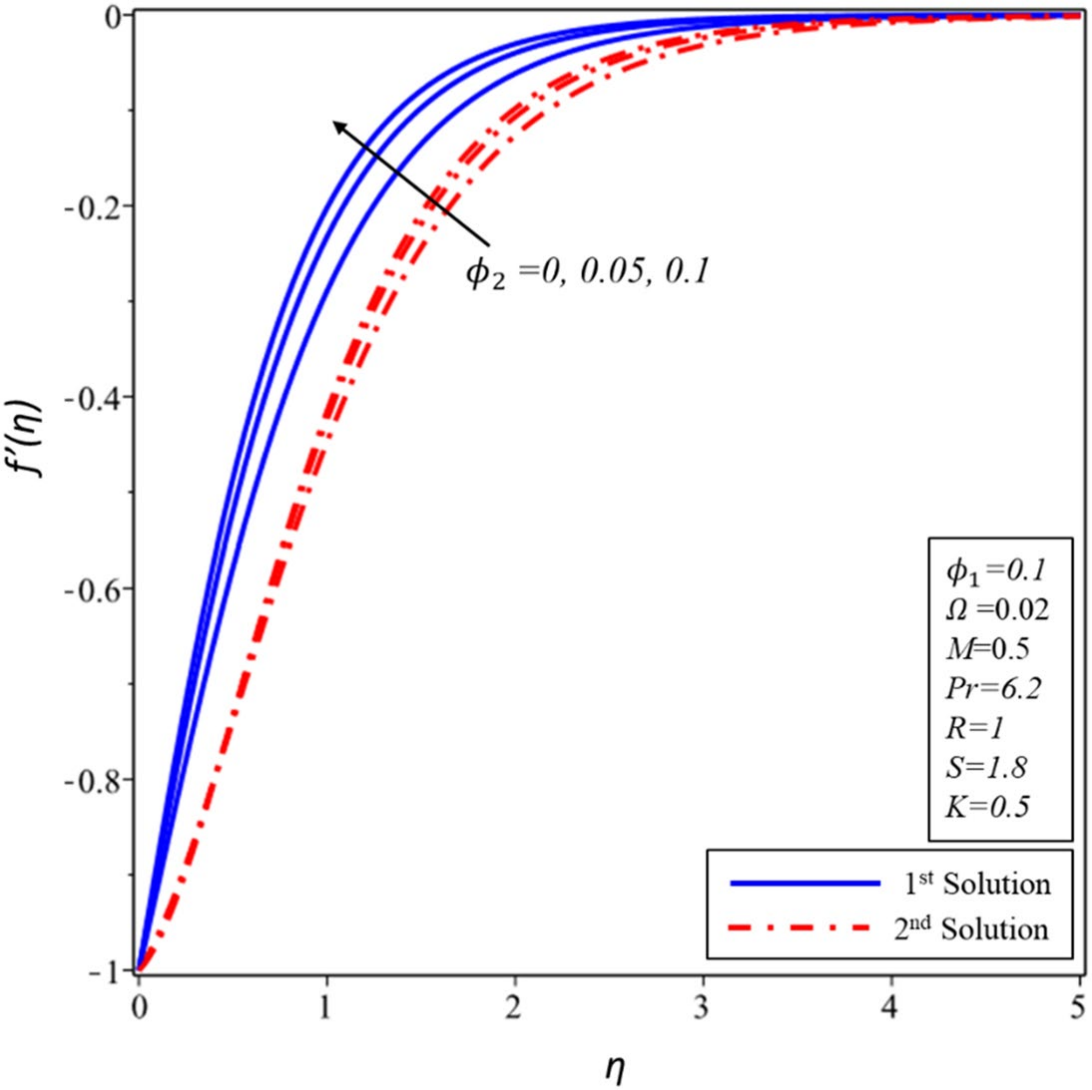
Variation of $f^{\prime}\left( \eta \right)$ for $\emptyset_{2}$ against $\eta$.

**Figure 15:**
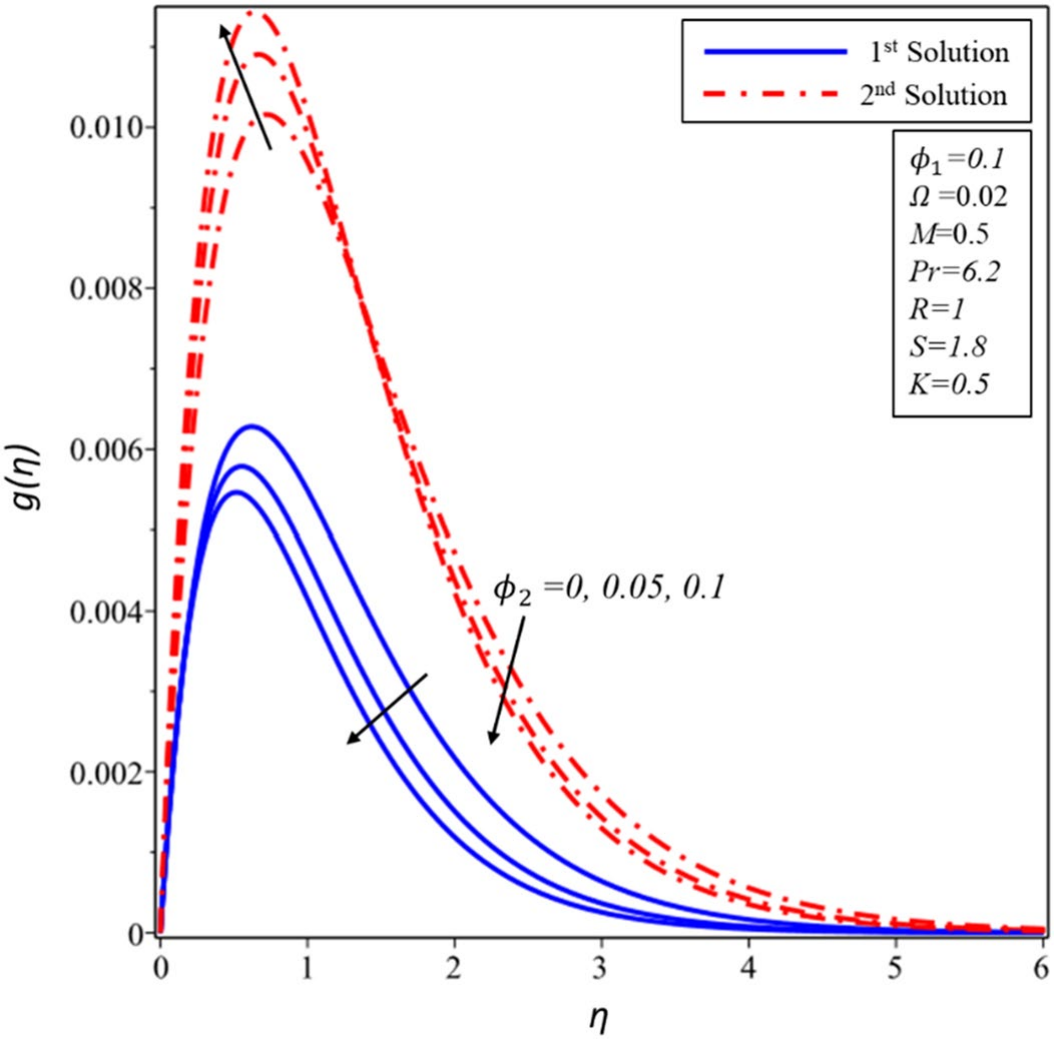
Variation of $g\left( \eta \right)$ for $\emptyset_{2}$ against $\eta$.

**Figure 16:**
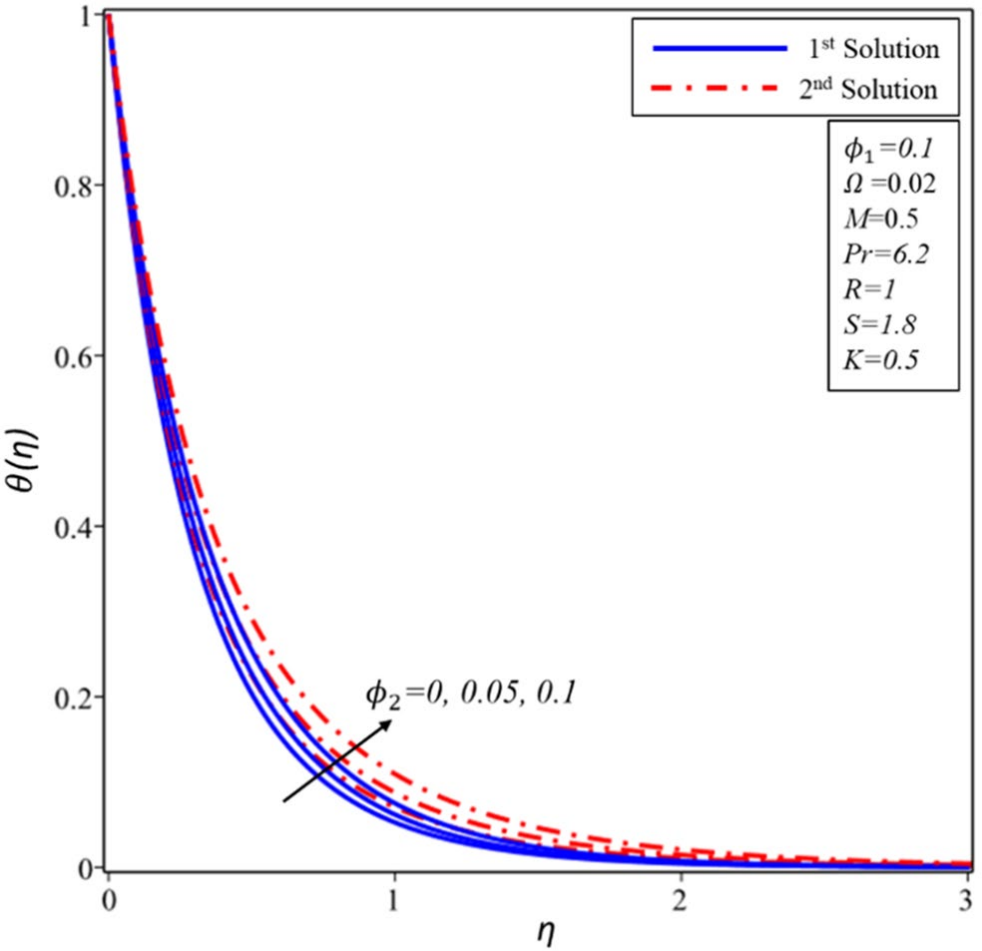
Variation of $\theta \left( \eta \right)$ for $\emptyset_{2}$ against $\eta$.

The effects of volume fraction of copper (${\mathrm{\varnothing }}_{2}$) are examined in Figures 14, 15 and 16 for the distributions of velocity $f^{\prime}(\eta )$, $g(\eta )$ and temperature $\theta (\eta )$. All profiles comply asymptotically with the BCs (10) and also it is noticed that there are two solutions. Figures 14 and 15 show that the thickness of hydrodynamic boundary layer declines with rising values of ${\mathrm{\varnothing }}_{2}$ for both solutions but it should be noted that dual nature exists in the second solution during the examination of $g(\eta )$. A special phenomenon is found for the thickness of thermal boundary layer where it enhances in both solutions as the magnitude of ${\mathrm{\varnothing }}_{2}$ rises (see Fig. 16). In contrast to the first solution, it should also be noticed that the second solution boundary layer has a greater thickness.

**Figure 17:**
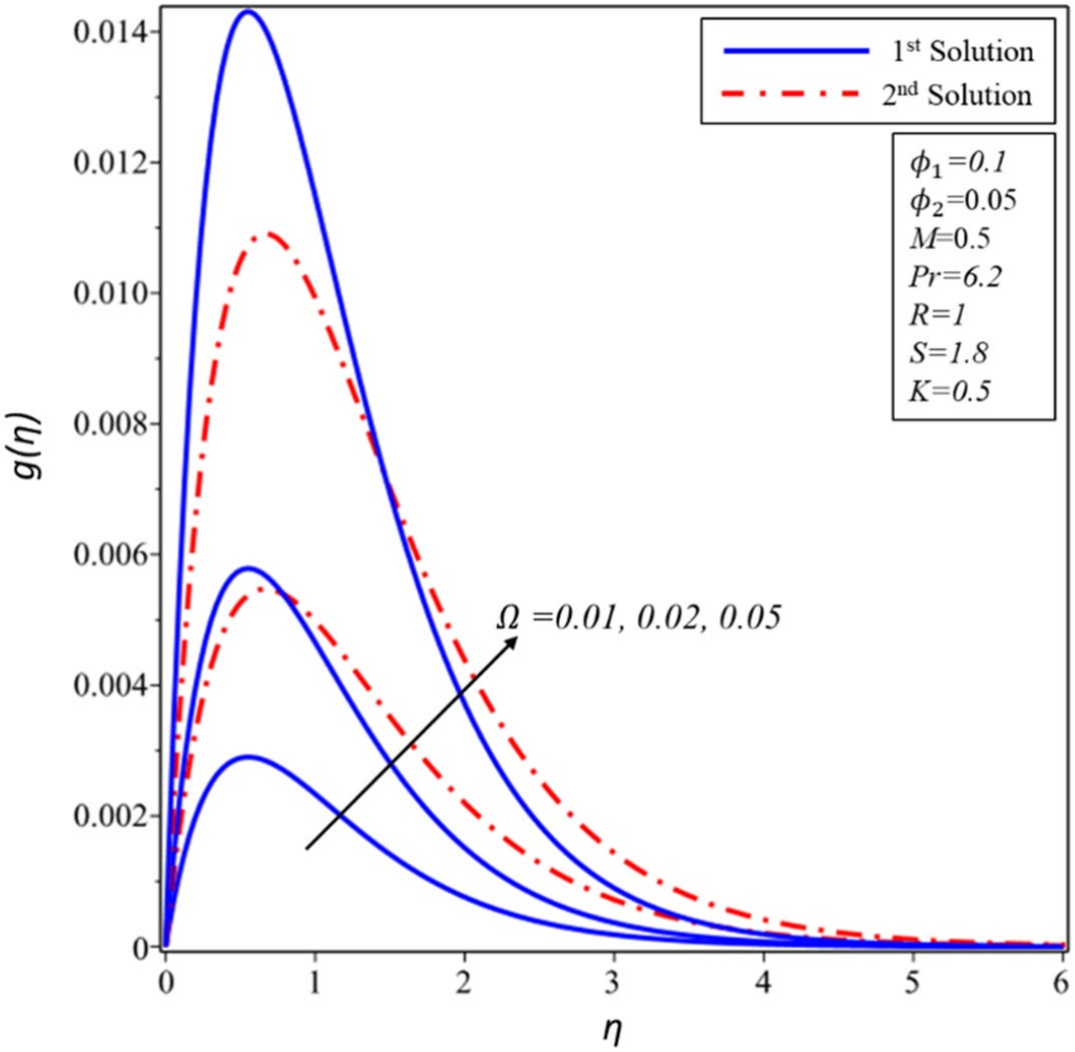
Variation of $g\left( \eta \right)$ for $\Omega$ against $\eta$.

Figure 17 illustrates that how the rotation parameter $\Omega$ effects on profiles of velocity $g(\eta )$. Increased rotation parameter $(\Omega )$ values result in a higher velocity of hybrid nanofluid and higher thickness of momentum layer in both solutions. It is also observed that only a single solution exists when $\Omega =0.05$. Larger values of $\Omega$ result in a higher rate of rotation in the second solution relative to the first solution. Consequently, the greater rotation results lead to the higher velocity of hybrid nanofluid and the higher thickness of momentum layer of all solutions.

**Figure 18:**
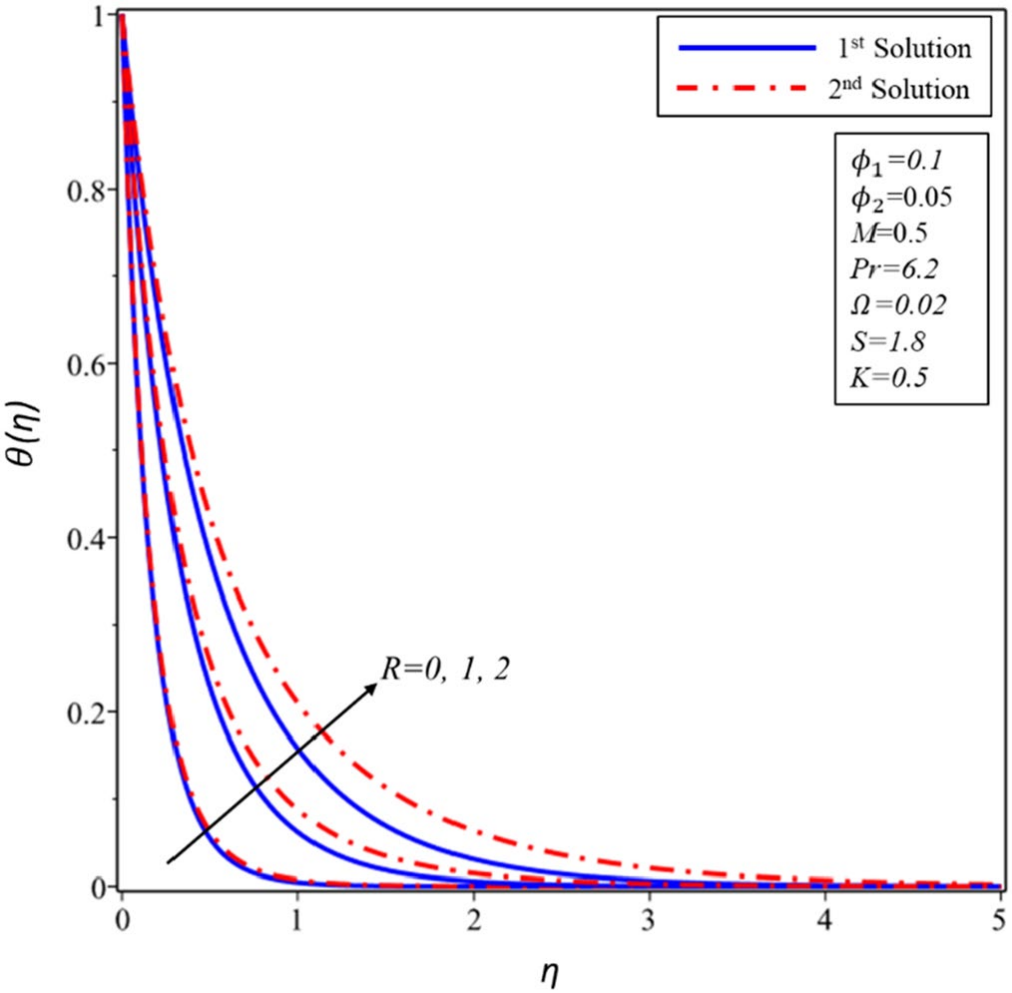
Variation of $\theta \left( \eta \right)$ for $R$ against $\eta$.

Figure 18 demonstrates the nature of the profiles of temperature for effects of radiation $R$. Such parameter appears only in the temperature Eq. (9) and is not attached to the momentum Eqs. (7–8); thus, the changes in $R$ do not induce any shifts in the profile of velocity. The thickness of boundary layers is shown to rise in all branches with an improvement in $R$ which suggests that the temperature gradient at the surface is smaller with higher $R$ magnitudes. Radiation parameter tests spread of the thermal radiation owing to the conduction heatwave. Higher values of $R,$ therefore indicate the predominance of thermal radiation on the conductions. As a consequence, because of $R$, the device emits a considerable volume of heat energy, which causes temperature to rise and indicates that the fluid temperature ($\theta (\eta )$) is increasing due to high radiation presence.

The governing Eqs. (26–29) were solved by using the bvp4c solver in Matlab software. The governing equations provide an infinite range of eigenvalues. Smallest negative eigenvalues: $\varepsilon <0$ implies that flow has an initial disruption development that may disrupt the flow and, ultimately, induce unstable flow. Besides that, the smallest positive eigenvalues; $\varepsilon >0$ shows that there is just an initial decay of disturbance, are showing stable flow. In this regard, Table 4 sets out the values of the smallest eigenvalue where it can be easily seen that the first solution is the stable one.

**Table 4:** Smallest eigenvalue $\varepsilon$ for different values of $K$ and $\phi_{2}$ when $S = 1.8, Pr = 6.2,\Omega = 0.02, M = 0.5, R = 1,F_{s} = 2, \phi_{1} = 0.1$.

$\phi_{2}$	$K$	$\varepsilon$	
		First Solution	Second Solution
0.0	1	0.9682	− 1.0613
	0.8	0.7493	− 0.8803
	0.6	0.4918	− 0.5099
	0.4499	0.0036	− 0.0042
0.02	1	0.8405	− 1.0424
	0.8	0.6570	− 0.8336
	0.6	0.4567	− 0.5146
	0.4332	0.0068	0.0359
0.05	1	0.7925	− 0.8352
	0.8	0.6431	− 0.6957
	0.6	0.3122	− 0.3438
	0.4033	0.0044	− 0.0107

## Conclusion

Darcy-Forchheimer 3D magnetized flow of $Cu+A{l}_{2}{O}_{3}+{H}_{2}O$ subject to rotating frame and radiation condition on the shrinking sheet was examined. The key results of the research discussed are set out below:

1. The presence of solutions relies on the suction parameter values.
2. The presence of dual solutions relies on the values of the porosity, coefficient of inertia, magnetic, and suction parameters for the specific values of the other applied parameters.
3. Mixing of copper nanoparticles in alumina water based nanofluid can adjust the profiles of temperature and velocity inside the boundary layer.
4. Higher values of $\Omega$ (${\phi }_{2}$) contribute to higher (lower) velocities in both solutions.
5. The increase in nanoparticle values expands the spectrum of solutions.
6. Decrease of heat transfer rate is noticed with an rise in the parameter of copper solid volume fraction.
7. The results of the stability analysis show that the first (second) solution is a (an) stable (unstable) solution.
